# Nucleomorph Small RNAs in Cryptophyte and Chlorarachniophyte Algae

**DOI:** 10.1093/gbe/evz064

**Published:** 2019-04-05

**Authors:** Anna K M Åsman, Bruce A Curtis, John M Archibald

**Affiliations:** 1 Department of Biochemistry and Molecular Biology, Dalhousie University, Nova Scotia, Canada; 2 Department of Molecular Sciences, Uppsala BioCenter, Swedish University of Agricultural Sciences and Linnean Center for Plant Biology, Uppsala, Sweden

**Keywords:** small RNAs, gene expression, nucleomorph, algae, cryptophytes, chlorarachniophytes

## Abstract

The regulation of gene expression and RNA maturation underlies fundamental processes such as cell homeostasis, development, and stress acclimation. The biogenesis and modification of RNA is tightly controlled by an array of regulatory RNAs and nucleic acid-binding proteins. While the role of small RNAs (sRNAs) in gene expression has been studied in-depth in select model organisms, little is known about sRNA biology across the eukaryotic tree of life. We used deep sequencing to explore the repertoires of sRNAs encoded by the miniaturized, endosymbiotically derived “nucleomorph” genomes of two single-celled algae, the cryptophyte *Guillardia theta* and the chlorarachniophyte *Bigelowiella natans*. A total of 32.3 and 35.3 million reads were generated from *G*. *theta* and *B*. *natans*, respectively. In *G. theta*, we identified nucleomorph U1, U2, and U4 spliceosomal small nuclear RNAs (snRNAs) as well as 11 C/D box small nucleolar RNAs (snoRNAs), five of which have potential plant and animal homologs. The snoRNAs are predicted to perform 2′-*O* methylation of rRNA (but not snRNA). In *B*. *natans*, we found the previously undetected 5S rRNA as well as six orphan sRNAs. Analysis of chlorarachniophyte snRNAs shed light on the removal of the miniature 18–21 nt introns found in *B*. *natans* nucleomorph genes. Neither of the nucleomorph genomes appears to encode RNA pseudouridylation machinery, and U5 snRNA cannot be found in the cryptophyte *G. theta*. Considering the central roles of U5 snRNA and RNA modifications in other organisms, cytoplasm-to-nucleomorph RNA shuttling in cryptophyte algae is a distinct possibility.

## Introduction

The plastids of modern-day algae and plants evolved from free-living cyanobacteria by endosymbiosis, that is, the incorporation of one cell inside another (Archibald 2015). While many algal lineages contain so-called “primary” plastids descended directly from cyanobacteria, this light-harvesting organelle has also spread horizontally between eukaryotic groups by “secondary” endosymbiosis, the uptake of a primary plastid-bearing alga by a nonphotosynthetic cell (Zimorski et al. 2014). Secondary endosymbiosis is known to have occurred several times during eukaryotic evolution, and has given rise to major algal groups that play fundamental roles in the global ecosystem, for example, diatoms, dinoflagellates, haptophytes, and brown algae (Not et al. 2012). Notably, in two secondarily evolved algal lineages, the cryptophytes and chlorarachniophytes, the nucleus of the primary algal endosymbiont persists in a miniaturized form called a “nucleomorph” (Tanifuji and Archibald 2014). Cryptophyte algae harbor a red-algal-derived plastid and nucleomorph while the chlorarachniophytes have a plastid–nucleomorph complex derived from a green alga (Archibald 2007). Sequenced nucleomorph genomes are a mere 370–700 kb in size and have 332–636 genes (Moore et al. 2012; Suzuki et al. 2015). In addition to nucleomorph genomes, complete nuclear, plastid, and mitochondrial genome sequences are available from the cryptophyte *Guillardia theta* and the chlorarachniophyte *Bigelowiella natans* (Curtis et al. 2012).

We are studying the roles of small RNAs (sRNAs) in nucleomorph genome biology. sRNAs are regulatory, sequence-specific guide molecules present in all domains of life (Künne et al. 2014). They are core components of ribonucleoprotein (RNP) complexes and use base-pairing interactions with target RNA or DNA to direct their associated proteins to their sites of activity. Splicing, the removal of intervening sequences (introns) from pre-mRNA, is an important aspect of eukaryotic gene expression. The molecular machine responsible for intron excision and exon ligation is the spliceosome (Will and Luhrmann 2011), an anciently evolved multimegadalton complex composed of five small nuclear RNAs (snRNAs) and a large number of proteins (∼100 in yeast and ∼200 in humans) (Fabrizio et al. 2009; Agafonov 2011). In addition to intron removal, splicing plays important roles in mRNA quality control and transcription elongation (Kornblihtt 2004; Fasken and Corbett 2009; Aslanzadeh et al. 2018). The nucleomorph genomes of secondary plastid-bearing algae have retained introns and genes for splicing machinery (Moore et al. 2012; Suzuki et al. 2015). Four chlorarachniophyte nucleomorph genomes have been sequenced to date, all of which contain a large number of exceedingly small introns (800–1,000 introns per genome, each 18–23 nt) (Suzuki et al. 2015). The sequenced nucleomorph genomes of cryptophyte algae contain far fewer introns (between 0 and 24; Moore et al. 2012).

The spliceosome is a ribozyme, with a core made up of five highly conserved snRNAs: U1, U2, U4, U5, and U6 (Will and Luhrmann 2011). The complexity of the nucleomorph spliceosomal machinery is unknown. The nucleomorph genome of the cryptophyte *G*. *theta* encodes only 15 proteins with predicted spliceosomal function (Douglas et al. 2001), and an as-yet undetermined number of snRNP proteins are encoded by the nuclear genome and imported posttranslationally. The *G. theta* nucleomorph genome contains 17 spliceosomal introns, which are 42–52 nt in size (Douglas et al. 2001). In the cryptophyte *Chroomonasmesostigmatica*, 24 nucleomorph introns were identified, ranging from 50 to 211 nt in length; this genome encodes 28 spliceosomal proteins (Moore et al. 2012). In contrast, *Cryptomonas paramecium* has only two introns (62 and 100 nt) and 17 genes for spliceosomal proteins (Tanifuji et al. 2011). The highest degree of spliceosome reduction is seen in the nucleomorph genome of *Hemiselmisandersenii*, where introns are completely absent and only four “spliceosomal” protein genes have been found (Lane et al. 2007).

Functional RNAs undergo several different types of nucleotide modifications as part of their maturation process. In rRNAs and snRNAs, ribose 2′-*O* methylation and isomerization of uridine to pseudouridine are the dominant modification types (Meier 2017; Sloan et al. 2017). These two posttranscriptional changes are catalyzed by C/D box and H/ACA snoRNPs, respectively, in which snoRNAs act as sequence-specific guides for protein-based catalysis. 2′-*O* methylation and pseudouridylation alter the biophysical properties of the targeted RNAs and confer increased ribosome and spliceosome stability (Meier 2017; Sloan et al. 2017). The modifications also play roles in the actual translation and splicing reactions, and act to regulate mRNA and protein levels (Krogh et al. 2016; Sloan et al. 2017; Zhao et al. 2018).

C/D box snoRNAs have conserved C/C′ (RUGAUGA) and D/D′ (CUGA) boxes, which base pair and fold into a K-turn (C/D) or a K-loop (C′/D′) structure (Massenet et al. 2017). The sequences upstream of the D and D′ boxes interact with the targeted RNA to guide methylation at the nucleotide that pairs exactly five residues from the D or D′ box (Kiss-László et al. 1998; Meier 2017). H/ACA snoRNAs typically consist of two stem loops separated by an H box (ANANNA) and end with a 3′ ACA motif.

The number of snRNAs and snoRNAs encoded by the *G*. *theta* nucleomorph genome is unclear. Douglas et al. (2001) originally reported five snoRNA genes (designated *snR1*-*5*) and one snRNA gene (*U6*) (Douglas et al. 2001). This nomenclature was later misinterpreted as suggesting the existence of a full set of snRNA genes (U1, U2, U4, U5, and U6) in the nucleomorph genome of this organism (Gilson et al. 2006). We have used sRNA sequencing and comparative RNomics to shed light on sRNA diversity in the nucleomorphs of cryptophytes and chlorarachniophytes. Specifically, we verify current nucleomorph sRNA sequence and structure predictions and identify novel sRNAs in both *G. theta* and *B. natans*. Numerous new intergenic sRNA-expressing loci were discovered, and several “missing” sRNAs were identified (cryptophyte U1, U2, and U4 snRNAs and chlorarachniophyte 5S rRNA). Six novel nucleomorph snoRNAs are described in *G*. *theta*, and a function is proposed for nucleomorph snoRNAs in rRNA 2′-*O* methylation.

## Materials and Methods

### Algal Cell Culturing

*G. theta* CCMP2712 and *B. natans* CCMP2755 were cultured in H2 medium and f/2-Si medium, respectively, at 22 °C under a 12:12 h light:dark regimen. Cell cultures were temporally synchronized following the procedures of Hirakawa et al. (2011) and harvested at a density of 0.5–0.8 × 10^6^ cells/ml 6 h after the onset of the light and dark phases. Duplicate (*G. theta*) or triplicate (*B. natans*) cultures were harvested for each treatment.

### sRNA Extraction and Sequencing

Low molecular weight RNA was extracted using the mirVana miRNA isolation kit using the protocol for enrichment of <200 nt RNAs provided by the manufacturer (Thermo Fisher Scientific, Waltham). For *G*. *theta* sRNA, an aliquot of one of the replicates was treated with Terminator Exonuclease (TE) using the standard protocol (Epicentre, Madison). The SMARTer smRNA-seq kit (TaKaRa, Kusatsu, Japan) was used to prepare barcoded sRNA-seq libraries (TruSeq HT indexes) in preparation for Illumina sequencing. Inserts were enriched for amplicons <150 bp using Agencourt AMPure XP beads (Beckman Coulter, Indianapolis). The *G*. *theta* and *B*. *natans* libraries were sequenced separately on a MiSeq instrument (Illumina, San Diego). The MiSeq reagent kit v2 (300 cycles) was used for *G*. *theta*, generating 35–70 nt reads. The *B*. *natans* samples were sequenced using the MiSeq reagent kit v3 (150 cycles), which generated 35–76 nt reads.

### sRNA Read Processing

Sequencing adaptors were removed using Cutadapt (Martin 2011). Mapping of sRNA reads to the *G. theta* nuclear, nucleomorph, plastid, and mitochondrial genomes was done using the “filter tool” and PatMaN in the UEA sRNA workbench v3.2 (Stocks et al. 2012). Reads were filtered if they were of low complexity, if they mapped to *G. theta* rRNA/tRNA, or if <16 nt in length. sRNA read mapping to the *B. natans* genomes was done using Bowtie2 v2.3.1 (Langmead and Salzberg 2012). Annotation of sRNA-expressing loci was done based on visual inspection of sRNA-seq peaks mapped to the *G*. *theta* and *B*. *natans* nucleomorph genomes. Distinct sRNA expression peaks were identified in intergenic regions, sometimes overlapping neighboring protein-coding genes at the 5′ or 3′ ends.

### snRNA Analyses

U4 snRNA was identified in the nucleomorph genomes of the chlorarachniophytes *B*. *natans*, *Amorphochlora amoebiformis*, *Lotharella vacuolata*, and *L*. *oceanica*, and the cryptophytes *G. theta* and *Ch.mesostigmatica* using the Infernal software package (Nawrocki and Eddy 2013). The Infernal tool cmbuild was used to create a covariance model (CM) based on the Rfam U4 sequence alignment (RF00015) (Kalvari et al. 2018). The CM was then applied in cmsearch to look for U4 in individual nucleomorph genomes. Searches for snRNAs U1 (RF00003), U2 (RF00004), U5 (RF00020), and U6 (RF00026) were done using the same method. RNA secondary structure predictions were done using RNAfold (Gruber et al. 2008).

Additional searches for *C. paramecium* U4 and *G*. *theta* U5 were done with the sequence pattern search tool PatScan (Dsouza et al. 1997). For U4, we scanned for sequences with the potential to interact with *C*. *paramecium* U6 snRNA (helix I: CATGCTAATCT, helix II: ATCCTTATACAGGGGC), allowing a maximum of two mismatches, two insertions, and two deletions. Potential U4 candidates were also required to fold into a hairpin between the two helices. The search pattern for U5 was the conserved loop I motif (TGCCTTTTACY; Y: pyrimidine) with up to six mismatches allowed.

In cases where CM searches were unsuccessful (e.g., in the identification of U1 and U2 candidates for *G*. *theta* and the minor spliceosomal components U11, U12, U4atac, and U6atac for *G. theta* and *B. natans*), snRNAs were identified manually based on 1) sRNA read peaks, 2) RNA structural folding, and 3) the presence of a conserved 5′ splice site (U1) or branch point (U2) interaction motif. Candidate snRNAs were required to fold into a U1 cloverleaf secondary structure or form typical U2 stem loops (SL) I/IIa/IIb/III.

### snoRNA Analyses

rRNA 2′-*O* methylation sites and guide snoRNAs from *Arabidopsis thaliana* and *Homo sapiens* were obtained from the snOPY database (Yoshihama et al. 2013). The program Plexy (Kehr et al. 2011) was employed for 2′-*O* methylation site prediction in nucleomorph 5.8S, small subunit (SSU), and large subunit (LSU) rRNAs and U1, U2, U4, U5, and U6 snRNAs. Searches for nucleomorph snRNA pseudouridylation sites were done using the snoGPS web server (Schattner 2004; http://lowelab.ucsc.edu/snoGPS/; last accessed March 27, 2019) and manual inspection.

Infernal was used to assess homology between the C/D box snoRNAs of nucleomorphs and those of plants and animals. The method was as described for U4 snRNA, except that CMs were built from alignments of snoRNA families (supplementary table 1, Supplementary Material online). Searches for snoRNA homologs in the diplomonad protist *Giardia lamblia* employed the genome of assemblage A, isolate WB, version 2013-02-08.

To locate homologs of *G*. *theta* nucleomorph snoRNAs in other cryptophytes, we identified the syntenic positions of the *G*. *theta* snoRNA-encoding loci in the nucleomorph genomes of *H.andersenii*, *C. paramecium*, and *Ch*. *mesostigmatica*. Syntenic sequences were then manually inspected for the presence of C/D box coding elements. Potential target sites in cryptophyte rRNAs were identified by alignment of nucleomorph, *Arabidopsis* and human rRNAs (supplementary table 2, Supplementary Material online) using Clustal Omega version 1.2.4.

### Identification of 5S rRNA

Chlorarachniophyte 5S rRNA was found by employing Infernal’s cmscan tool to search the nucleomorph genomes of *B*. *natans*, *L. vacuolata*, *L. oceanica*, and *A. amoebiformis* against the Rfam CM database (Nawrocki and Eddy 2013; Kalvari et al. 2018).

### Analysis of RNP Protein Homologs

BLASTp and tBLASTn were used to search for nucleomorph homologs of protein components of the spliceosome, the C/D box snoRNP and the H/ACA box snoRNP. Homologs from various organisms were used as queries including red algal sequences to help identify homologs in cryptophyte nucleomorph genomes. Reciprocal BLAST searches and HMM analysis (https://www.ebi.ac.uk/Tools/hmmer/; last accessed March 27, 2019) were used to validate the *G*. *theta* and *B*. *natans* protein candidates. To analyze the potential targeting of nuclear-encoded proteins to the nucleomorph, N-terminal signal and target peptide sequences were predicted using SignalP 3.0 and TargetP 1.1 (Emanuelsson et al. 2007). To account for possible errors in pre-existing gene models, alternative start codons and translated N-terminal sequences were identified and analyzed manually.

### Analysis of Differential Gene Expression under Light and Dark Growth

Identification of sRNA-expressing loci and delineation of sRNA 5′/3′ ends were done by manual inspection of sRNA reads mapped to the nucleomorph genomes. The sRNA reads from each species were pooled and *de novo* assembled using Trinity v2.5.0 (Haas et al. 2013). Within the Trinity framework, Bowtie2 v2.3.3.1 (Langmead and Salzberg 2012) was used to map the sRNA reads to nucleomorph sRNA loci. Transcript abundance was estimated using RSEM v1.2.3 (Li and Dewey 2011) and converted to TMM matrices, which were used with the Bioconductor package EdgerR (Robinson et al. 2010) for differential expression analyses. sRNAs were considered as differentially expressed if |log_2_-fold change| ≥ 1.3 and false discovery rate (FDR)<0.05 (Benjamini and Hochberg 1995). Within-sample, across-transcript expression level variation was assessed by analyzing TPM (transcripts per million) values (not cross-sample normalized).

## Results and Discussion

Small RNA (sRNA) libraries were sequenced from duplicate (*G*. *theta*) or triplicate (*B*. *natans*) algal cultures collected in light and darkness. For *G. theta*, we also sequenced libraries prepared from TE-treated sRNAs. This enzymatic treatment degrades 5′-monophosphate sRNAs, such as fragments of ribosomal RNAs (rRNAs), and enriches for 5′-capped transcripts, for example, snRNAs and snoRNAs. Illumina sequencing generated 32,295,326 and 35,290,584 raw reads from *G*. *theta* and *B*. *natans*, respectively, which were mapped to the nuclear, nucleomorph, mitochondrial, and plastid genomes of the two organisms (supplementary tables 3 and 4, Supplementary Material online).

Focusing on nucleomorph sRNAs, we searched for “hotspots” of mapped sRNA reads in the nucleomorph genomes of the two organisms. To qualify as a candidate sRNA gene, we required that a locus show sRNA accumulation in all sequenced libraries. We found that all such loci were intergenic, that is, no sRNA genes were located fully inside a protein/rRNA/tRNA-encoding gene. Excluding known rRNA- and tRNA genes, 21 and 14 sRNA-producing loci were identified in the *G*. *theta* and *B*. *natans* nucleomorph genomes, respectively.

### A Near-Complete Set of Spliceosomal RNAs in the Cryptophyte Nucleomorph

Only one spliceosomal RNA gene, *U6 snRNA*, has been identified in cryptophyte nucleomorph genomes sequenced thus far (Douglas et al. 2001; Tanifuji et al. 2011; Moore et al. 2012). While it is conceivable that the nucleomorph spliceosome functions with a reduced set of snRNAs, loss of four of the five snRNAs (U1, U2, U4, and U5) otherwise conserved across eukaryotic evolution would be an unprecedented example of spliceosome reduction. Indeed, extensive snRNA gene loss in some or all nucleomorphs is difficult to envisage mechanistically, considering the central role of snRNAs at the core of the spliceosomal machinery. It is possible that the nucleomorph uses snRNAs encoded by the host genome, that is, snRNAs from genes transferred from the nucleomorph to the host genome through endosymbiotic gene transfer (EGT). A third possibility is that genes for U1, U2, U4, and U5 snRNAs are in fact present in nucleomorph genomes but that their genes have not been detected due to high sequence divergence.

In an effort to identify the missing snRNAs in the *G*. *theta* nucleomorph, we employed a search pipeline consisting of four bioinformatic steps. We first searched for snRNA candidates using Infernal (Nawrocki and Eddy 2013), a tool that uses CM building to search for structural RNAs based on both primary sequence and secondary structure. We then sought support for the candidate snRNA-encoding loci in our sRNA-seq data and analyzed predicted secondary structures of the snRNA candidates. Finally, we assessed the ability of the snRNAs to engage in base-pairing interactions with the previously characterized nucleomorph U6 snRNA. We identified strong candidates for U1, U2, and U4 snRNAs, and confirmed the previously annotated U6 snRNA locus (supplementary table 5, Supplementary Material online). The four snRNAs were represented by sequence reads in all six libraries, including those generated from TE-treated samples. This indicates that the nucleomorph snRNAs do not have monophosphate 5′ ends, but rather possess classical snRNA 5′-end structures, that is, a 2,2,7-trimethylguanosine cap (U1, U2, U4) or a gamma-monomethyl group (snRNA U6) (Hinas et al. 2006).

**Fig. 1.**
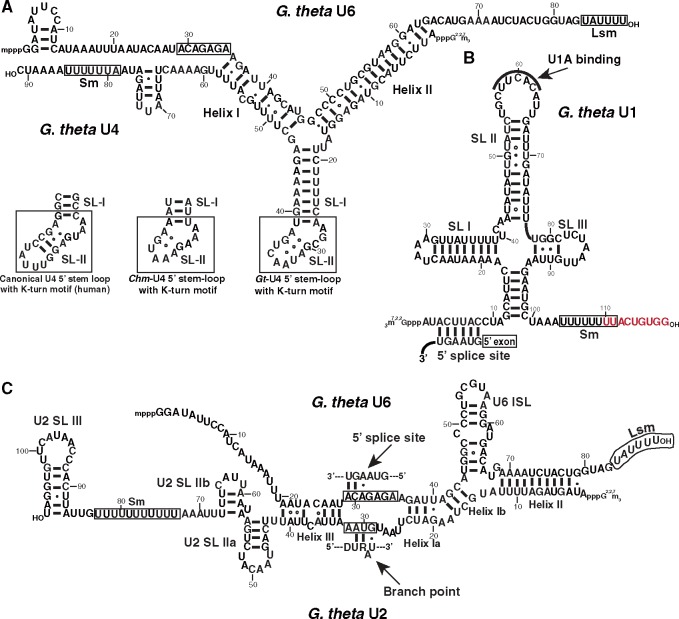
—Predicted secondary structures and intermolecular interactions of *Guillardia theta* nucleomorph spliceosomal RNAs. The 3′-proximal Sm and Lsm sites are boxed in each snRNA. (*A*) U4–U6 interaction. The putative K-turn structures of U4 snRNA from human and the cryptophytes *Chroomonas mesostigmatica* and *G*. *theta* are shown. The cryptophyte K-turn SL II contains two conserved sheared G–A base pairs, but is otherwise shorter than canonical SL II. The 5′ splice site-binding region of U6 is boxed. (*B*) U1 snRNA. The 5′ splice site-binding region and the U1A-interacting motif are indicated. Letters in red overlap with the downstream protein-coding gene. (*C*) U2–U6 interaction. The 5′ splice site-binding region of U6 and the branch point interaction site of U2 are boxed (D; A/G/U).

***Guillardia*** ***theta* U1 snRNA.** The *G. theta* nucleomorph-encoded snRNA U1 adopts a typical U1 cloverleaf-like secondary structure and has a conserved 5′ splice site recognition sequence “ACUUAC” at its 5′ end (Konarska 1998; Pomeranz Krummel et al. 2009) (fig. 1*B*). This motif exhibits perfect complementarity to the 5′ splice site of the nucleomorph genome’s 17 introns (GUAAGU) (Douglas et al. 2001). The sRNA read coverage ends just seven nucleotides downstream of the Sm site, resulting in a proposed secondary structure that lacks the generally conserved stem-loop (SL) IV. Interestingly, SL IV is also missing in the U1 snRNA of the parasite *Giardia lamblia*, a diplomonad protist with a highly reduced genome (Hudson et al. 2012).

In humans, two paralogous proteins, U1A and U2B, bind respectively to SL II of U1 snRNA and SL IV of U2 snRNA (Williams and Hall 2011). In addition, U1 SL I is bound by protein U1-70k (Surowy et al. 1989). In *G. theta*, a recognizable binding site for protein U1A is found in SL II of U1, but no binding site for protein U1-70k is apparent. Curiously, genes for U1A/U2B and U1-70k are missing in the *G*. *theta* nucleomorph genome. The nuclear genome encodes three U1A/U2B proteins (XM_005827036, XM_005827131, XM_005838408). One of them (XM_005827036) is distantly related to the other two, and has the potential for a signal peptide upstream and in-frame of the currently annotated gene model. This N-terminal motif could thus conceivably target the gene product to the nucleomorph and the periplastidial compartment (PPC) in which it resides (supplementary data 1, Supplementary Material online) (Curtis et al. 2012). However, whether XM_005827036 is derived from the nucleomorph via EGT or is a repurposed and retargeted host protein is currently unclear; like many genes in the *G. theta* genome (Curtis et al. 2012), the phylogenetic history of this locus is ambiguous (data not shown).

***Guillardia*** ***theta* U2 snRNA**. A candidate for U2 snRNA was identified by manually searching our intergenic sRNA loci for sequence complementarity to snRNA U6. We identified a sequence able to extensively base pair with U6 by formation of three canonical U2:U6 intramolecular RNA helices (fig. 1*C*). This candidate sequence folds into a typical U2 secondary structure and contains a canonical branch-point (BP) interacting motif. Only a weak BP recognition motif can be identified in the *G*. *theta* nucleomorph introns; instead of a classical “YURAC” motif (where A is the BP adenosine), we identified a “DURAU” pentanucleotide close to the intron 3′ end in 12 of 17 introns (D = A/G/U). The remaining five introns have as-yet unidentified BP sequences. The “DURAU” motif is located 2–6 nt from the intron 3′ splice site, in the majority of cases 2–3 nt from it (fig. 1*C*). Notably, the *G*. *theta* nucleomorph U2 snRNA does not have a 3′ SL IV, which is known to be the interaction site of protein U2B. This suggests that nuclear-encoded U1A/U2A, if indeed targeted to the nucleomorph, interacts specifically with U1 SL II.

***Guillardia*** ***theta* U4 snRNA**. A U4 snRNA-like sequence was identified in *G. theta* by CM analysis and verified by manual inspection of sRNA-seq reads. The putative U4 snRNA has the potential to form extensive base-pairing interactions with snRNA U6, which results in the formation of two characteristic U4/U6 intermolecular helices (fig. 1*A*). Between the two helices, U4 is predicted to fold into a typical 5′ SL. The secondary structure of canonical U4 snRNA (e.g., from human) has a well-characterized and functionally important kink-turn (K-turn) motif in the 5′ SL. This structure is the binding site of protein Snu13, a critical RNP assembly factor shared between the U4 and C/D box snoRNPs (Watkins et al. 2002). A Snu13 homolog is encoded by the *G*. *theta* nucleomorph, indicating that the U4 K-turn could be a binding platform for this protein.

The *G*. *theta* U4 snRNA K-turn is shorter than the corresponding structure in humans and yeast (Nottrott et al. 1999): SL II is only 3 bp long and lacks Watson–Crick base pairs. To investigate the potential significance of the shortened K-turn SL II, we searched for U4 homologs in the nucleomorph genomes of three other cryptophytes, *H. andersenii*, *C*. *paramecium*, and *Ch*. *mesostigmatica*. No genes for *G*. *theta* U4 were detected in syntenic regions of the three other sequenced nucleomorph genomes, so we applied CM analysis and sequence pattern searches instead of synteny (Dsouza et al. 1997; Nawrocki and Eddy 2013). *Hemiselmisandersenii* has previously been reported to lack introns and a splicing apparatus, and as expected, we did not find any snRNA genes in its nucleomorph genome. Perhaps more surprisingly, a *U4* gene could not be found in the genome of *C*. *paramecium* either. This indicates that the *C*. *paramecium* gene for *U4* is either too divergent to be detected, or has been transferred to the nuclear genome by EGT. In *Ch*. *mesostigmatica*, U4 was found to have only 2 bp in K-turn SL II, very similar to the situation in *G*. *theta* (fig. 1*A*). For reference, U4 K-turn SL II in the red alga *Cyanidioschyzon merolae* is only slightly larger, at 4 bp long (Stark et al. 2015).

### No U5 snRNA Is Detected in *G*. *theta*

Previous experiments have shown that U5 snRNP is indispensable for the formation of a functional spliceosome, and U5 snRNA is the only RNA component common to the major U2-dependent and minor U12-dependent spliceosome (Nancollis et al. 2013). U5 snRNA tethers the 5′ exon to the spliceosome after the first catalytic step and aligns the two exons for the second transesterification reaction (Will and Luhrmann 2011). A highly conserved motif in U5 snRNA loop I plays a critical role in this process, as it interacts directly with both the 5′ and the 3′ exon to assure correct alignment for ligation during step two of the splicing reaction (Sontheimer and Steitz 1993; Nancollis et al. 2013).

CM analysis did not identify a U5-like sequence in the nucleomorph genomes of any of the four cryptophytes. The absence of a clear U5 snRNA candidate is different from the case in chlorarachniophytes, where a U5-encoding gene is found in all published nucleomorph genomes (Suzuki et al. 2015). Considering the central role of the U5 snRNP in the splicing process, absence of U5 snRNA from the cryptophyte nucleomorph appears unlikely. An as-yet undetected and highly divergent nucleomorph *U5 snRNA* gene is perhaps more plausible. Alternatively, the nucleomorph might utilize a posttranscriptionally imported, nuclear*-*encoded, U5 snRNA. We could identify three *U5 snRNA* genes in the host nuclear genome, but the three sequences differ at only one position, which precludes easy assignment of one of them as an obvious nucleomorph-to-nucleus EGT. It is nevertheless conceivable that one of the nuclear U5 snRNAs is nucleomorph-localized, either as a stand-alone nucleomorph spliceosome component, or as a dual-targeted nuclear/nucleomorph snRNA.

### snoRNA-Guided Methylation of *G. theta* Nucleomorph rRNA

Very little is known about ribosome biogenesis and maturation in the nucleomorph and PPC. RNA modifications have so far not been characterized in nucleomorphs, and the 2′-*O* methylation and pseudouridylation status of nucleomorph RNAs is unknown. The former is likely to occur, since initial sequencing and annotation of the *G*. *theta* nucleomorph genome identified five 2′-*O* methylation guide snoRNAs (designated snR1-5 by Douglas et al. 2001 and “mistakenly”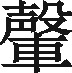 interpreted as including U1, U2, U4, and U5 snRNAs by Gilson et al. 2006). These five snoRNA loci (renamed GtNM-R5-9 herein) are highly expressed (supplementary table 6, Supplementary Material online); in fact, GtNM-R5, GtNM-R7, and GtNM-R8 are among the top four most abundant transcripts out of 21 identified sRNAs in terms of TPM. The snoRNAs were detected in the libraries from TE-treated and nontreated samples, indicating that these RNAs, just like typical eukaryotic snoRNAs, are 5′ capped (Massenet et al. 2017).

**Fig. 2.**
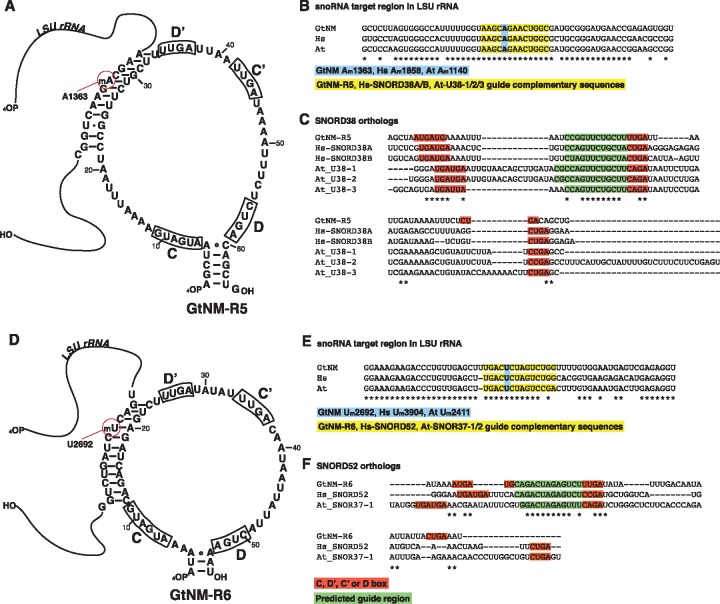
—The *Guillardia theta* C/D box snoRNAs GtNM-R5 and GtNM-R6 have homologs in *Arabidopsis* and human. (*A*) Secondary structure model of GtNM-R5 and its interaction with nucleomorph LSU rRNA. The predicted D′ guide region and the 2′-*O* methylation target position A1363 are indicated. (*B*) GtNM-R5, human SNORD38 and *Arabidopsis* U38 have identical LSU targets. (*C*) Sequences of SNORD38 homologs. Guide regions in human and *Arabidopsis* were obtained from the snOPY database (Yoshihama et al. 2013). The 2′-*O* methylation guide region (green) is conserved between nucleomorph GtNM-R5 and the human and *Arabidopsis* SNORD38 homologs. (*D*) Secondary structure model of GtNM-R6 and its interaction with nucleomorph LSU rRNA. The predicted D′ guide region and the 2′-*O* methylation target position U2692 are indicated. (*E*) GtNM-R6, human SNORD52, and the *Arabidopsis* homolog SNOR37 have highly similar LSU targets. (*F*) Sequences of SNORD52 homologs. Guide regions in human and *Arabidopsis* were obtained from the snOPY database (Yoshihama et al. 2013). The 2′-*O* methylation guide region (green) is conserved between nucleomorph GtNM-R6 and the human and *Arabidopsis* SNORD52 homologs. (C), (D′), (C′), and (D) boxes are shown in red background. At: *Arabidopsis thaliana* Gt: *Guillardia theta*, Hs: *Homo sapiens*, NM: nucleomorph.

To identify C/D boxes in GtNM-R5-9, we manually scanned the five sequences for conserved box motifs (Massenet et al. 2017). This led to the identification of near-canonical C, D, and D′ boxes in all five snoRNAs, and a shortened C′ motif in GtNM-R5-8 (fig. 2*A* and *D*, supplementary table 5, Supplementary Material online). The latter motif is located at a distance from the D′ box conforming to the D′–C′ spacing originally described by Kiss-László et al. (1998).

The snoRNA target prediction tool Plexy (Kehr et al. 2011) identified a large number of potential rRNA interaction sites in GtNM-R5-9. Given that rRNA modifications occur in highly conserved core regions of SSU and LSU rRNAs (Bachellerie 1995; Sloan et al. 2017), it is possible to align the nucleomorph rRNAs with rRNAs from *Arabidopsis* and human, and to compare the predicted nucleomorph methylation positions with well-characterized plant and animal methylation patterns. Two sites in SSU rRNA and eight sites in LSU rRNA were found to overlap with experimentally verified 2′-*O* methylation sites in human or plant rRNAs (table 1). The five snoRNAs conform to the universal “+5 rule” common to eukaryotic and archaeal C/D box snoRNA guides; modification at the nucleotide interacting five positions upstream from the D/D′ box (Kiss-László et al. 1996; Dennis et al. 2001). Two methylation sites were predicted in 5.8S rRNA, neither of which overlap with human or plant 2′-*O* methylation targets.

**Table 1:** Predicted rRNA Targets of *Guillardia theta* Nucleomorph C/D Box snoRNAs

snoRNA^a^	Box Guide	Nucleomorph rRNA Target	Human rRNA Position/snoRNA^b^	*Arabidopsis* rRNA Position/snoRNA^b^
**GtNM-R5**	D′	SSU-A1040	SSU-G867/SNORD98	—
		LSU-A1363	LSU-A1858/**SNORD38A, SNORD38B**	LSU-A1140/**U38-1, U38-2, U38-3**
			LSU-A2774/SNORD99	
				—
		LSU-A2070		
**GtNM-R6**	D′	LSU-U2692	28S-U3904/**SNORD52**	LSU-U2411/**SNOR37-1, SNOR37-**2
GtNM-R7	D′	LSU-C3263	LSU-C4506/SNORD35A, SNORD35B	LSU-C2949/U35
**GtNM-R8**	D	LSU-A2526	LSU-A3739/**SNORD46**	—
**GtNM-R9**	D	SSU-C613	SSU-C462/**SNORD14A, SNORD14B**	SSU-C416/**U14a, U14b, U14c, U14d**
			LSU-C2791/SNORD55	
				LSU-C1850/U55, SNOR15
		LSU-C2087	—	LSU-C2340/SNOR77Y-1
		LSU-C2620	LSU-C3848/SNORD53	LSU-C2355/U53, SNOR37-1, SNOR37-2
		LSU-C2635		
**GtNM-R10**^c^	D′	LSU-G3119	LSU-G4362/**SNORD1**	LSU-G2805/**SNOR38Y-2**
		LSU-G1139	LSU-G1612/SNORD80	—
		LSU-G2393	—	LSU-G2114/U60.1F
**GtNM-R11**^c^	D′	SSU-A162	SSU-A166/SNORD44	SSU-A162/SNOR18a, SNOR18b
				LSU-A2936/**U29**
		LSU-A3250	LSU-A4493/**SNORD29**	
GtNM-R12^c^	D	SSU-G1666	SSU-G1490/SNORD25	SSU-G1431/SNOR19-1, SNOR19-2
				LSU-G1845 SNOR59a, SNOR59b
		LSU-G2082	—	
GtNM-R13^c^	D	LSU-A1038	LSU-A1511/SNORD51	LSU-A814/U51a, U51b
GtNM-R14^c^	D′	LSU-G2150	—	LSU-G1913/U40-2
GtNM-R15^c^	D′	LSU-A2551	LSU-A3764/SNORD15A, SNORD15B	LSU-A2271/U15-1a, U15-1b, U15-2

Note.—Human and *Arabidopsis* snoRNAs and their corresponding SSU and LSU rRNA positions are shown.

^a^ snoRNAs **in bold** were identified as likely homologs in the current study. Two snoRNAs were considered as homologs if they share the same rRNA target site and were predicted homologs by CM analysis.

^b^ rRNA modification positions and snoRNA guide identities in human and *Arabidopsis* were obtained from the snoRNA orthological gene database (Yoshihama et al. 2013).

^c^ Discovered in this study.

### The C/D snoRNA Machinery Is Conserved among Cryptophytes

To gather additional evidence for the validity of snoRNAs GtNM-R5-9, we searched for homologs in the nucleomorph genomes of *C*. *paramecium*, *H*. *andersenii*, and *Ch*. *mesostigmatica*. First, candidate snoRNAs were identified in each cryptophyte by synteny analysis. Next, the identified candidates were searched manually for motifs resembling the box elements of the putative *G*. *theta* RNAs: C box AUGAUG(A), D box CUGA, C′ box (U/A)UGA, and D′ box (U/A)UGA. Potential homologs of GtNM-R8-9 were found in all three nucleomorph genomes, whereas prospective NM-R7 homologs were identified in *H*. *andersenii* and *Ch*. *mesostigmatica*. A putative NM-R6 could only be found in the *H*. *andersenii* nucleomorph genome and no sequence resembling GtNM-R5 was identified in any of the three additional cryptophytes.

The C box consensus is preserved in the four cryptophytes, with the exception of *C*. *paramecium* NM-R9: CUGAUG(A). A canonical D box (CUGA) was found in all snoRNAs. The snoRNA guide regions are conserved between the organisms, but an overall high degree of sequence variation is observed outside of the box elements and the guides (supplementary fig. 1, Supplementary Material online). Regardless, SSU and LSU rRNAs are most likely snoRNP targets in the nucleomorphs of all four species; target predictions with the *C*. *paramecium*, *H*. *andersenii*, and *Ch*. *mesostigmatica* snoRNA candidates identified the same *Arabidopsis*/human-conserved rRNA modification positions as in *G*. *theta*.

All four cryptophyte nucleomorph genomes encode the core C/D box snoRNP protein components Fibrillarin, Snu13, Nop56, and Nop58 (Massenet et al. 2017). Of the H/ACA snoRNP, only the catalytic component Cbf5 (pseudouridine synthase) is predicted. The nuclear genome of *G*. *theta* has genes for the three missing H/ACA components, Nhp2, Gar1, and Nop10, although the encoded proteins lack obvious N-terminal nucleomorph targeting peptides (Gould et al. 2006; Gruber et al. 2007). At present, we can conclude that the rRNAs of the cryptophyte nucleomorph are likely 2′-*O* methylated, but their pseudouridylation status is uncertain.

### Plant and Human Homologs of Cryptophyte snoRNAs

CM analysis predicted GtNM-R5 to be homologous to SNORD38, a broadly distributed snoRNA with family members in plants, animals, and fungi (Yoshihama et al. 2013). GtNM-R5 is predicted to methylate LSU-A1363, whose homologous positions are validated methylation targets of SNORD38 in both *Arabidopsis* and human (fig. 2*B*and table 1). GtNM-R5 and SNORD38 have identical LSU rRNA targets and highly similar guides, but display sequence divergence outside of these conserved regions (fig. 2*B* and *C*).

A potential snoRNA homolog of GtNM-R6 was also identified; both rRNA sequence alignment and CM analysis suggest a shared ancestry between GtNM-R6 and metazoan SNORD52 (fig. 2*E* and *F*). The predicted LSU rRNA target site of GtNM-R6 is shares a target site with human SNORD52 and its *Arabidopsis* homolog, SNOR37 (fig. 2*E*and table 1). Notably, human SNORD52 and GtNM-R6 have identical guide sequences and rRNA targets.

GtNM*-*R8 shares target site with human SNORD46, and the two snoRNAs have identical guide sequences. However, CM analysis did not recognize homology between the two RNAs. GtNM*-*R8 is instead predicted as a homolog of *Gi*. *lamblia* GlsR7, a snoRNA that has itself been predicted to be a homolog of SNORD46 (Yang et al. 2005). GlsR7, GtNM*-*R8, and SNORD46 have highly similar guides and target the same site in LSU rRNA.

**Fig. 3.**
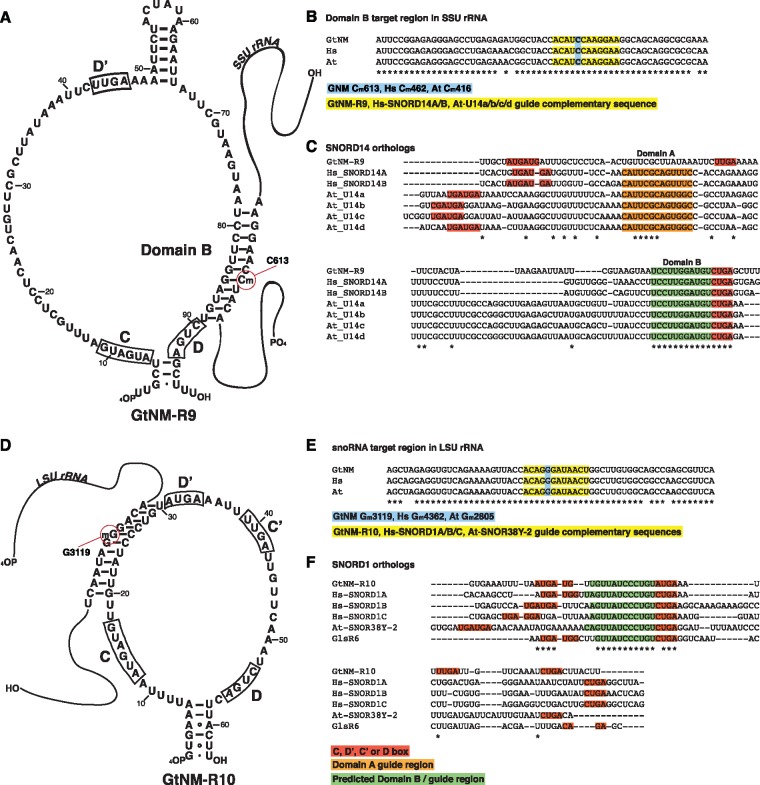
—Homologs of *Guillardia theta* C/D box snoRNAs GtNM-R9 and GtNM-R10 are found in human and *Arabidopsis*. (*A*) Secondary structure model of GtNM-R9 and its interaction with nucleomorph SSU rRNA. The predicted Domain B and the 2′-*O* methylation target position C613 are highlighted. (*B*) GtNM-R9, human SNORD14 and *Arabidopsis* U14 have identical SSU targets. (*C*) Sequences of SNORD14 homologs. Guide regions in human and *Arabidopsis* were obtained from the snOPY database (Yoshihama et al. 2013). Human and *Arabidopsis* SNORD14 homologs contain a Domain A (orange), guiding SSU rRNA processing. Domain B (green) guides 2′-*O* methylation and is conserved between nucleomorph GtNM-R9 and human and *Arabidopsis* SNORD14 homologs. (*D*) Secondary structure model of GtNM-R10 and its interaction with nucleomorph LSU rRNA. The predicted D′ guide region and the 2′-*O* methylation target position G3119 are indicated. (*E*) GtNM-R10, human SNORD1, and the *Arabidopsis* homolog SNOR38Y-2 share LSU target. (*F*) Sequences of SNORD1 homologs. Guide regions in human and *Arabidopsis* were obtained from the snOPY database (Yoshihama et al. 2013). The 2′-*O* methylation guide region (green) is conserved between nucleomorph GtNM-R10 and the human and *Arabidopsis* SNORD1 homologs. (C), (D′), (C′), and (D) boxes are shown in red background. At: *Arabidopsis thaliana* Gt: *Guillardia theta*, Hs: *Homo sapiens*, NM: nucleomorph.

We predicted GtNM-R9 to be a homolog of SNORD14, an unusually long snoRNA that so far has been identified in plants, opisthokonts, ciliates, and excavates (Andersen and Nielsen 2012; Moore and Russell 2012; Kalvari et al. 2018). Unique among C/D box snoRNAs, SNORD14 has dual functions, guiding both processing and 2′-*O* methylation of SSU rRNA. The two activities are carried out by two separate segments of the snoRNA, domain A (pre-rRNA cleavage) and domain B (rRNA methylation) (Moore and Russell 2012). In line with the extended length of typical SNORD14 sequences (Andersen and Nielsen 2012; Moore and Russell 2012; Kalvari et al. 2018), GtNM-R9 is the longest of the nucleomorph snoRNAs identified herein (fig. 3*A*).

SNORD14 methylation functionality appears to be preserved in GtNM-R9, as it shares predicted SSU rRNA 2′-*O* methylation targets with *Arabidopsis*, human, yeast, and rice (fig. 3*B* and *C*, table 1) (Moore and Russell 2012; Yoshihama et al. 2013). However, domain A functionality may have been lost in the nucleomorph homolog, because GtNM-R9 does not contain a guide sequence complementary to the conserved SSU rRNA processing motif. Domain A is not recognizable in any other nucleomorph snoRNA either, which rules out the possibility that guiding of SSU rRNA cleavage has been taken over by another snoRNA (at least those we identified). Loss of SNORD14 domain A and retention of domain B is the reverse of the situation in *Euglena*, *Tetrahymena*, and Diptera, where Domain B is missing, but the processing function has been preserved (Andersen and Nielsen 2012; Moore and Russell 2012).

Given the known distribution of dual-activity SNORD14 homologs among the eukaryotic supergroups, the ancestral snoRNA present in the last eukaryotic common ancestor (LECA) most probably had both functions (Moore and Russell 2012). Several studies have reported the absence of domain B (Yuan et al. 2003; Andersen and Nielsen 2012; Moore and Russell 2012), and with our observation that GtNM-R9 lacks function A, it is clear that the two domains have been differentially lost during eukaryotic evolution. In yeast, deletion of domain A is lethal, while the methylation function is dispensable (Jarmolowski et al. 1990). The nucleomorph rRNA maturation pathway is unknown, but it is conceivable that 18S rRNA is produced as its own transcriptional unit, sidestepping the need for snoRNA-guided rRNA cleavage. Involvement of nuclear-encoded SNORD14 in the process is another possibility, since two *snord14* genes with A + B domains are identifiable in the *G*. *theta* nuclear genome.

### Six Novel C/D Box snoRNAs in the Cryptophyte Nucleomorph

The snoRNA orthological gene database (snOPY) lists 220 snoRNAs in *Arabidopsis*, 760 in humans, and 77 in yeast (Yoshihama et al. 2013). Obligate parasites such as the diplomonad *Giardia* and Microsporidia possess significantly smaller sets of snoRNAs: 21 in *Gi. lamblia* and 10 in *Encephalitozoon cuniculi* (Li et al. 2012; Belkorchia et al. 2017). As is the case for nucleomorphs, these organisms have small genome sizes and rely on their hosts for many metabolic functions.

The presence of a complete set of C/D box snoRNP proteins in *G. theta* raises the possibility of additional snoRNAs beyond GtNM-R5-9 in the cryptophyte nucleomorph. CM analysis with RNA families SNORD1 and SNORD29 identified two novel potential C/D box snoRNAs with similar expression levels as snoRNAs GtNM-R5-9 (supplementary table 6, Supplementary Material online). The SNORD1 family is highly conserved, with clearly identifiable homologs in *Arabidopsis*, rice, yeast, and human. This is in contrast to the many lineage-specific snoRNAs that have been discovered in eukaryotes (Chen 2003; Yoshihama et al. 2013; Kalvari et al. 2018). An in silico target search with GtNM-R10 (fig. 3*D*) predicted three positions to be shared with plant and animal methylation sites (table 1), one of which is targeted by SNORD1 (Yoshihama et al. 2013). The nucleomorph, plant, and animal snoRNAs have very similar guide regions and identical LSU target sequences (fig. 3*E* and *F*). The putative homology between GtNM-R10 and SNORD1 is strengthened by the fact that *Gi*. *lamblia* snoRNA GlsR6 appears to be homologous to both sequences (supplementary table 1, Supplementary Material online). GtNM-R10 and GlsR6 share targets with human and *Arabidopsis* SNORD1 in a highly conserved region of LSU rRNA (Yang et al. 2005; Yoshihama et al. 2013).

GtNM-R11 is a potential nucleomorph homolog of SNORD29, with which it shares methylation target sites in both *Arabidopsis* and human (table 1). We identified two truncated versions of GtNM-R11 (34 nt) on *G. theta* chromosome 3; the two pseudogenes lack box C′ and box D and show no evidence of expression in our sRNA read data.

An additional four novel snoRNAs, all with noncanonical box C and D motifs, were discovered by manual inspection of the nucleomorph sRNA loci (table 1). GtNM-R12-14 have nucleotide substitutions at the last position of box C and the first position of box D (AUGAUU and AUGA). In eukaryotic and archaeal snoRNAs, the C and D boxes interact to fold into a K-turn, the formation of which is critical for C/D box snoRNP assembly and 2′-*O* methylation activity structure (Klein et al. 2001; Henras et al. 2004). Importantly, the two C/D box substitutions in GtNM-R12-14 are compensatory, so that a Watson–Crick base pair can still be formed between the 3′ nt of box C and the 5′ nt of box D. Mutation analyses of archaeal snoRNA showed that single substitutions at the exact box C/D sites that are changed in GtNM-R12-14 led to an altered structure of the core C/D RNA motif and decreased affinity for protein L7 (the archaeal homolog of eukaryotic Snu13) (Kuhn et al. 2002). Restoration of Watson–Crick base pairing through simultaneous mutation of both positions recovered L7 binding strength and RNA folding. Thus, K-turn assembly in GtNM-R12-14 should be unaffected by the nucleotide changes seen in the C/D box.

GtNM-R15 has a canonical nucleomorph box D (CUGA) but two substitutions in box C (GUGAAG; supplementary table 5, Supplementary Material online). The C box 5′ G conforms to the eukaryotic consensus (5′ purine) and is located at an unpaired position in the K-turn internal loop. The second substitution replaces a U–U pair with a Watson–Crick A–U pair at the base of stem II. Kuhn et al. (2002) showed that this U–U to A–U substitution had no effect on L7 protein binding to the K-turn. In contrast, introducing a C–U pair significantly reduced L7 affinity to the RNA. One of the defining features of the K-turn is the presence of two sheared G–A base pairs at the base of stem II (Klein et al. 2001). These two G–A base pairs are retained in the C and D boxes of all 11 *G. theta* nucleomorph snoRNAs (GtNM-R5-15).

A search for rRNA complementarity in GtNM-R12-15 identified a number of potential 2′-*O* methylation target sites, five of which are shared with *Arabidopsis* and human snoRNAs (table 1). Convergent evolution might however account for these shared targets, because homology between the nucleomorph, animal, and plant snoRNAs was not readily apparent.

### Conserved snoRNAs and rRNA Methylation Sites

Based on the prediction of 20 shared snoRNA target sites in plants, animals, and the cryptophyte nucleomorph, we can infer likely methylation of at least 20 nucleomorph rRNA nucleotides. Additional positions might be modified, especially if nuclear-encoded C/D box snoRNAs were to be imported into the nucleomorph. Profiling of ribose methylations by, for example, 2OMe-seq (Incarnato et al. 2017) in the nucleomorph would help to determine the total number of 2′-*O* methylated positions.

A previous study of the evolution of eukaryotic snoRNAs inferred at least 25 snoRNA families to have been present in LECA (Hoeppner and Poole 2012). This conclusion was based on the distribution of snoRNA homologs among the major eukaryotic supergroups and the conservation of experimentally verified rRNA modification sites in plants, animals, and yeast. Three of these 25 putative LECA snoRNA families—SNORD14, SNORD29, and SNORD38—have potential homologs in *G*. *theta*’s nucleomorph (GtNM-R9, GtNM-R11, and GtNM-R5, respectively). These three nucleomorph snoRNAs thus have a deep evolutionary ancestry. The remaining eight nucleomorph snoRNAs appear to be the result of lineage-specific gains before or during the uptake of the red alga whose nucleus eventually became the nucleomorph.

### Cryptophyte Nucleomorph snoRNAs Do Not Appear to Target snRNAs

Methylation targets were predicted in *G*. *theta*’s spliceosomal RNAs using the same target prediction pipeline that was applied to the rRNAs. A few interaction sites could be predicted between GtNMR5-15 and the snRNAs, but none overlapped with experimentally characterized human or *Arabidopsis* snoRNA targets. The nucleomorph C/D box snoRNAs GtNM-R5-15 therefore seem to act specifically on SSU and LSU rRNAs. We do however note that the nucleomorph snRNAs have diverged considerably in sequence from their *Arabidopsis* and human counterparts, which complicates identification of homologous nucleotide positions.

Pseudouridylation, the other major type of modification of snRNA, stabilizes spliceosome structure and facilitates snRNA–protein interactions (Donmez 2004; Meier 2017). Human snRNAs are particularly heavily modified; U2, for example, has 13 pseudouridines and 10 2′-*O* methylated nucleotides (Donmez 2004). Since no pseudouridylation machinery appears to be present in the nucleomorph, the question of if, and how, the nucleomorph spliceosome functions without snRNA 2′-*O* methylation and pseudouridylation is presently unclear.

### Orphan sRNAs in the Cryptophyte Nucleomorph

Our sRNA-seq analysis identified a total of 21 sRNA loci in the cryptophyte nucleomorph. Setting aside the previously described sRNAs (Douglas et al. 2001) and our newly identified snRNAs and snoRNAs, this leaves six novel putative loci. The six sRNAs of unknown function (supplementary fig. 2, Supplementary Material online) are all located in unannotated intergenic regions. GtNM-R16 and GtNM-R18 are completely intergenically encoded, while the other four sRNAs overlap a flanking protein-coding gene at the 5′ or 3′ end (13–43 nt overlap; supplementary table 5, Supplementary Material online). Overlapping transcription is not unique to noncoding RNAs, as it has been reported to occur at high levels for nucleomorph mRNAs (Williams et al. 2005; Tanifuji et al. 2014b).

Transcription of novel sRNA GtNM-R20 (supplementary fig. 2, Supplementary Material online) appears to start ∼20 nt into the upstream gene *hira*, and the 130-nt intergenic space between *hira* and *rps6* is completely covered with sRNA-seq reads. Our attempts to identifying GtNM-R20 homologs in the nucleomorph genomes of *H*. *andersenii*, *C*. *paramecium*, and *Ch*. *mesostigmatica* (Lane et al. 2007; Tanifuji et al. 2011; Moore et al. 2012) were unsuccessful; no sequence or structure conservation was apparent in the syntenic regions of any of the four nucleomorph genomes.

GtNM-R21 (supplementary fig. 2, Supplementary Material online) is expressed at a similarly high level as the most abundant snoRNAs (supplementary table 6, Supplementary Material online). The bulk of the GtNM-R21 coding region (130 nt) is located in the intergenic space between *kin(snf1)* and *orf714*, but 43 nt overlap the downstream gene. Transcript overlap in the 3′ direction conforms with the observations of nucleomorph mRNAs by Williams et al. (2005), who reported 31 cases of transcriptional overlap at the 3′ end, and 3 cases of 5′ overlap. Our sRNA read data supports 3′ overlapping transcription for nine RNAs and only one instance of 5′ transcriptional overlap (supplementary table 5, Supplementary Material online). A similar observation was made for *B*. *natans* nucleomorph sRNAs (supplementary table 7, Supplementary Material online).

### Single-Copy *5S rDNA* Gene in the Chlorarachniophyte Nucleomorph Genome

Initial analysis of the *B*. *natans* nucleomorph genome reported the absence of *5S rDNA* from the subtelomeric rDNA locus (Gilson et al. 2006). In contrast, the *G*. *theta* nucleomorph encodes a complete set of eukaryotic rRNAs (5S-LSU-5.8S-SSU) at each of its six chromosome termini (Douglas et al. 2001). Analyzing our mapped sRNA reads from *B*. *natans*, we identified a sRNA-expressing locus in close proximity to the rDNA repeat. This sRNA, transcribed from the unannotated region between *dnaK* and *rpl3*, was identified as 5S rRNA by both homology and CM analysis (supplementary table 7, Supplementary Material online). No *5S rDNA*-like sequence was identified at the five *dnaK* pseudogene loci or anywhere else in the genome. Thus, *5S rDNA* is a single-copy gene in *B*. *natans*, in contrast to the *5.8S*, *SSU*, and *LSU* rDNAs.

The discovery of a gene encoding 5S rRNA in *B*. *natans* is consistent with the presence of the 5S rRNP genes *rpl5* and *rpl11*. Genes for Rpl5 and Rpl11 are present also in the nucleomorph genomes of three other chlorarachniophytes, *L*. *vacuolata*, *L*. *oceanica*, and *A*. *amoebiformis* (Suzuki et al. 2015), and we identified previously unannotated *5S rDNA* in these three genomes as well (supplementary data 1, Supplementary Material online). The ribosome of the chlorarachniophyte nucleomorph thus appears to be composed solely of nucleomorph-encoded rRNAs, although a number of ribosomal protein genes are missing from the nucleomorph genome; these proteins have either been lost or are nucleus-encoded (Curtis et al. 2012).

### Chlorarachniophyte Nucleomorph Spliceosomal RNAs

The 373-kb nucleomorph genome of *B*. *natans* contains a total of 865 introns (18–21 nt in size) (Gilson et al. 2006; Suzuki et al. 2015), substantially more than seen in *G*. *theta* (17 introns in total) and other cryptophytes. This corresponds to an intron density of ∼3 introns/kb of coding sequence, less than in vertebrates and land plants, but more than in, for example, prasinophyte green algae, oomycetes, diatoms, and ascomycete fungi (Csuros et al. 2011). What follows is a description of the snRNAs encoded by the *B. natans* nucleomorph genome and what these data tell us about splicing in the chlorarachniophyte nucleomorph.

**Fig. 4.**
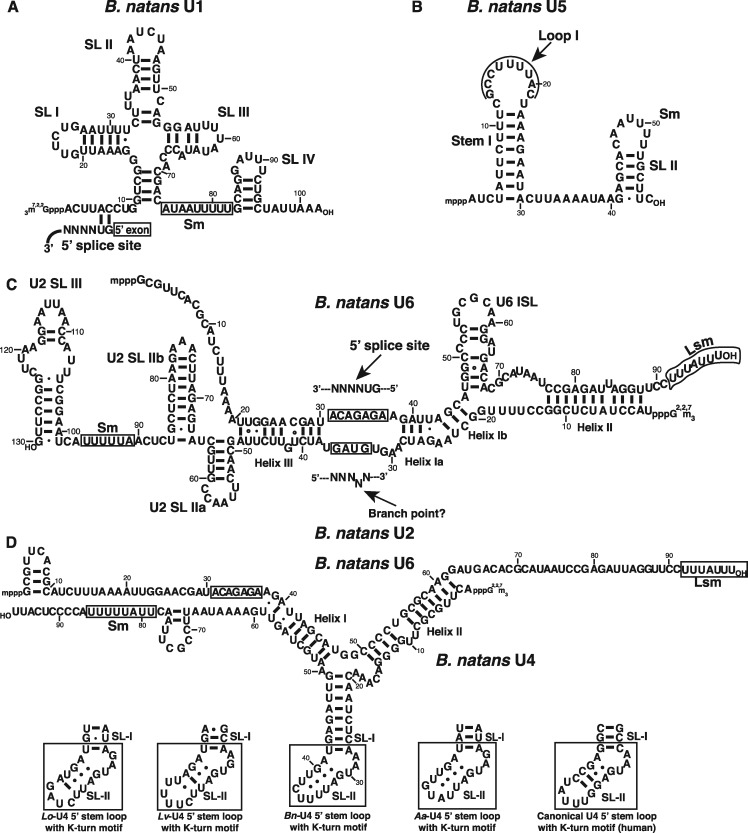
—Predicted secondary structures and intermolecular interactions of *Bigelowiella natans* nucleomorph spliceosomal RNAs. The 3′-proximal Sm and Lsm sites are boxed in each snRNA. (*A*) U1 snRNA. The 5′ splice site-binding region is indicated. (*B*) U5 snRNA. Stem I contains a highly conserved loop I motif. (*C*) U2–U6 interaction. The 5′ splice site-binding region of U6 and the branch point interaction site of U2 are boxed. (*D*) U4–U6 interaction. The putative K-turn structures of U4 snRNA from *Lotharella oceanica*, *L*. *vacuolata*, *B*. *natans*, *Amorphochlora amoebiformis*, and human are shown. The chlorarachniophyte K-turn SL II contains a reduced number of Watson–Crick base pairs. The 5′ splice site-binding region of U6 is boxed.

**Chlorarachniophyte snRNA U4.** Previous nucleomorph genome sequencing efforts reported the absence of U4 snRNA from four chlorarachniophyte species (Gilson et al. 2006; Tanifuji et al. 2014a; Suzuki et al. 2015). *U4 snRNA* was however predicted bioinformatically in the *B*. *natans* nucleomorph genome by Dávila López et al. (2008), and we can confirm the presence of *U4 snRNA* in all four sequenced chlorarachniophyte genomes. The putative U4 is capable of forming extensive base-pairing interactions with *B*. *natans* U6 snRNA (fig. 4*D*), and has ample sRNA-seq support. Just like the cryptophyte U4, the 5′ SL of *B*. *natans* U4 forms no Watson–Crick base pairs at the 3′ side of the sheared G–A pairs. Formation of K-turn SL II therefore relies on the comparably weaker base-pairing interactions provided by a doublet of U–U pairs. Such noncanonical U–U base pairs are common in RNA, especially in duplex junctions and loops, and stacking of two consecutive U–U pairs is known to increase RNA duplex stability (Sheng et al. 2013). The SL II K-turn in the *A*. *amoebiformis* U4 has the same two nonstandard U–U base pairs as in *B*. *natans*, while *L. vacuolata* U4 has a standard A–U pair and a U–U pair (fig. 4*D*). SL II of *L*. *oceanica* U4 has the A–U and U–U bases in the reverse order compared with *L. vacuolata*. Conservation of the U–U pair in SL II underscores the functional importance of this non-Watson–Crick interaction in the K-turn of chlorarachniophyte U4.

In the classical U4/U6.U5 tri-snRNP, the K-turn is bound by Snu13. This protein is required for correct folding of the K-turn and for snRNP assembly (Omer et al. 2006; McPhee et al. 2014). A single-copy *Snu13* is found in the *B*. *natans* nucleomorph genome. A recent study investigated the role of the base pair adjacent to the sheared G–A doublet and found that this pair determines whether or not the K-turn forms spontaneously or requires the binding of Snu13 (McPhee et al. 2014). Human, yeast, and *C*. *merolae* U4 snRNA have a G–C pair at this position (Nottrott et al. 1999; Stark et al. 2015), whereas the chlorarachniophyte U4 contains either a U–U or an A–U pair. Importantly, all three base combinations lead to an inability of the K-turn to fold unless bound by protein Snu13 (McPhee et al. 2014). This requirement for protein binding most likely ensures that the U4/U6.U5 complex is assembled at the correct stage of the multistep splicing reaction.

***Bigelowiella*** ***natans* U1 snRNA.** Our sRNA expression data support the existence of a U1 snRNA that is significantly shorter (102 nt) than the originally annotated sequence (153 nt) and the Rfam U1 consensus (166 nt) (Gilson et al. 2006; Kalvari et al. 2018). Despite its reduced size, the 102-nt version identified here is predicted to fold into an archetypical U1 cloverleaf conformation composed of three SL structures and a highly shortened 3′ SL IV (fig. 4*A*).

Apart from the splice donor GU dinucleotide, the introns of the chlorarachniophyte nucleomorph do not contain any obvious 5′ exon border motifs (Gilson et al. 2006; Slamovits and Keeling 2009). Nonetheless, we identified a fully conserved 5′ splice site recognition motif in *B*. *natans* U1 (fig. 4*A*), perhaps suggestive of an additional role of the U1 snRNA 5′ motif beyond intron binding. No binding site for protein U1A is apparent in *B*. *natans* U1, which agrees with the absence of any genes for U1A/U2B in sequenced chlorarachniophyte nucleomorph genomes. The nuclear genome contains one copy each of *u1a* and *u2b*, neither of which encodes a protein with an obvious PPC/nucleomorph targeting motif.

***Bigelowiella*** ***natans* U2 snRNA.** The Rfam reference U2 snRNA is 193 nt long, while the published *B*. *natans* nucleomorph U2 is 141 nt (Gilson et al. 2006). Eleven nt at the 3′ end lack support from the RNA-seq data generated in our study; our updated U2 snRNA is thus 130 nt in length. This sequence contains typical U2 snRNA structural features such as SL I, IIa, IIb, and III (fig. 4*C*). It lacks SL IV, consistent with the absence of a gene for U1A/U2B. While chlorarachniophyte nucleomorph introns lack obvious BP interaction motifs (Gilson et al. 2006; Slamovits and Keeling 2009), we identified a BP interaction sequence (GUAG) in the *B*. *natans* U2 snRNA. The sequence surrounding the GUAG motif is highly conserved, including several uracils that are converted to pseudouracils in human U2 snRNA (Donmez 2004).

***Bigelowiella*** ***natans* U5 snRNA.** The U5 snRNA predicted from the nucleomorph genome is 120 nt long, close to the consensus Rfam length of 116 nt (Gilson et al. 2006; Kalvari et al. 2018). In contrast, our sRNA expression data suggest a significantly shorter U5, only 58 nt long. Just like the canonical U5 snRNA in other organisms, the reduced U5 folds into two SL structures but they are both considerably stunted (fig. 4*B*). The published *B*. *natans* U5 contains an unusually large loop I, and its secondary structure places SL I far from its typical position at the 5′ end. In our minimal U5 snRNA, SL I has a perfect Rfam consensus loop I motif (YGCCUUUUACY) (O’Keefe 2002; Kalvari et al. 2018). This loop is known to bind the central U5 snRNP protein Prp8, of which a homolog is encoded in the *B*. *natans* nucleomorph genome.

***Bigelowiella*** ***natans* U6 snRNA.** Our sRNA-seq data support the existence of a 98-nt long U6 snRNA, which is only two nucleotides shorter than the published sequence and very close to the canonical 104-nt U6 snRNA (fig. 4*C* and *D*) (Gilson et al. 2006; Kalvari et al. 2018). The tiny introns of the *B*. *natans* nucleomorph do not contain an identifiable 5′ splice site recognition motif, but as with U1, the U6 sequence has retained an archetypical U6 snRNA 5′ splice site binding sequence (ACAGAGA).

### Contrasting Spliceosome Evolution in Red and Green Algal-Derived Nucleomorphs

No snRNAs of the minor spliceosome were identified in the nucleomorphs of *G*. *theta* or *B*. *natans*, which is in line with the presence of GU-AG intron boundaries in both genomes (Douglas et al. 2001). Splicing has been reported to occur in >90% of transcripts in the *B*. *natans* nucleomorph, which is similar to estimates in eukaryotic model organisms (Gilson et al. 2006; Wong et al. 2018). The process of nucleomorph intron removal thus appears to be efficient in *B*. *natans*, supported by the presence of a full set of snRNAs. By contrast, intron retention is prevalent in the nucleomorph of the cryptophyte *G*. *theta* (Wong et al. 2018), where the spliceosomal machinery appears to be less complete in terms of the number of nucleomorph-encoded proteins (Gilson et al. 2006) and snRNAs. We found no trace of U5 snRNA in the cryptophyte nucleomorph, but curiously, the genome encodes the major U5 snRNP proteins Prp8, Brr2, and Snu114 (Douglas et al. 2001). This is at odds with the loss of U5 snRNA from the nucleomorph spliceosome, and makes import of a nuclear-encoded U5 a distinct possibility. Given that U5 loop I has been deemed essential for the splicing reaction (Frank et al. 1994; O’Keefe 1998; Nancollis et al. 2013), it is conceivable that this element and its functionality has been transferred to another nucleomorph snRNA. We could not however find any loop I-like motif in any of the nucleomorph snRNAs, snoRNAs, or orphan sRNAs examined herein. Experimental validation, for example, by immunoprecipitation of the U5 snRNP complex, will be required to identify the potential snRNA included in the cryptophyte U5 snRNP.

Spliceosomal introns and snRNAs have been investigated in a few additional reduced genomes. Similar to the cryptophyte nucleomorph (Wong et al. 2018), microsporidian parasites have low splicing efficiency and display variation in intron density and spliceosome completeness between species (Desjardins et al. 2015; Belkorchia et al. 2017). The red alga *C. merolae* is missing U1 snRNA but has an unusually large U5 (450 nt) (Stark et al. 2015). Similar to the case in *G*. *theta*’s nucleomorph, the diplomonads *Gi. lamblia* and *Spironucleus vortens* apparently lack U5 snRNA (Hudson 2014). This snRNA has however been identified in the related species *S*. *salmonicida*. It is thus possible that more refined bioinformatic searches will reveal U5 snRNA in *Gi*. *lamblia*, *S. vortens*, and the *G*. *theta* nucleomorph.

Our sRNA sequencing revealed the presence of shortened versions of U1 and U5 snRNAs expressed from the *B*. *natans* nucleomorph. The diminutive sizes of U1 and U5 parallel the very short introns in the chlorarachniophyte nucleomorph (18–23 nt). Remarkably, many important structural features are retained in the miniscule U1 and U5 snRNAs. U1 snRNA has a typical U1 cloverleaf structure, including SL IV, which is missing from *G*. *theta*’s longer U1 snRNA (figs. 1*B* and 4*A*). The essential loop I of U5 snRNA (Frank et al. 1994; O’Keefe 1998; Nancollis et al. 2013) is perfectly conserved in *B*. *natans* (fig. 4*B*), as is the overall RNA structure. The main difference is the length of the 5′ stem, which is radically reduced relative to its counterpart in model organisms. In yeast, deletion analysis has showed that the minimal functional U5 consists of loop I, an internal loop in the 5′ stem plus the Sm binding site (Frank et al. 1994). The *B*. *natans* U5 snRNA 5′ stem lacks the internal loop, but otherwise resembles the yeast minimal U5 in both structure and size. It is possible that the very short 5′ stem in the nucleomorph U5 has coevolved with the small introns in these highly reduced genomes.

### Orphan sRNAs in the *B*. *natans* Nucleomorph

After identification of 5S rRNA and U4 snRNA, eight additional expressed sRNAs of unknown function were apparent. One of them, BnNM-R7 (supplementary table 7, Supplementary Material online), encodes previously unannotated tRNA-Phe(GAA) (Gilson et al. 2006); this is the first phenylalanine-specifying tRNA to be reported from the *B*. *natans* nucleomorph.

Of the remaining *B. natans* sRNAs, five (BnNM-R8-R12) contain potential box C and D motifs and have lengths and secondary structures reminiscent of the nucleomorph C/D box snoRNAs described earlier for *G*. *theta*. Target site prediction identified potential interactions with SSU and LSU rRNAs for these sRNAs, but only one predicted modification site is the same as an experimentally verified 2′-*O* methylation position (supplementary fig. 3, Supplementary Material online). Consistent with this apparent lack of target site conservation, we did not find any obvious plant or human snoRNA homologs of the five *B*. *natans* snoRNA candidates; we thus classified them as orphan sRNAs (supplementary fig. 4 and table 7, Supplementary Material online).

Curiously, the *B*. *natans* nucleomorph genome encodes three out of four core C/D snoRNP proteins: Fibrillarin, Snu13-like, and Nop56/Nop58 (Gilson et al. 2006). One of the Nop56 and Nop58 paralogs appears to be missing, but we could not confidently tell which one. Possibly, nucleomorph Nop56/58 functions as a homodimer, as is the case of the archaeal snoRNP (Dennis et al. 2001). Notably, the nucleomorphs of *L. vacuolata* and *L*. *oceanica* each have two *nop56/58* genes (Tanifuji et al. 2014a; Suzuki et al. 2015). In any case, the presence of proteins of the C/D snoRNP, but no snoRNAs, suggests that chlorarachniophyte nucleomorph RNA 2′-*O* methylation might be guided by nuclear-encoded snoRNAs.

We did not identify any H/ACA boxes among the *B*. *natans* orphan sRNAs, which is in line with the complete lack of any genes for H/ACA snoRNP proteins in the *B*. *natans* nucleomorph genome (Gilson et al. 2006).

### Potential Functions of Nucleomorph Orphan sRNAs

Organellar genomes are ubiquitously transcribed, which is a probable source of regulatory RNAs (Tanifuji et al. 2014b; Sanitá Lima and Smith 2017). Mining of public RNA-seq data revealed that over 85% of plastid, nucleomorph, and mitochondrial genomes are transcriptionally active (Sanitá Lima and Smith 2017). In some cases, including the nucleomorph genomes of the cryptophytes *H*. *andersenii* and *C. paramecium*, ∼99% of the genome is represented at the RNA level.

In the present study, a number of expressed sRNAs of unknown function were discovered. These orphan sRNAs may have lineage- or nucleomorph-specific functions yet to be discovered. Alternatively, they might carry out roles similar to those described for sRNAs in model organisms but have diverged to the point that homology cannot be established. In light of pervasive and overlapping nucleomorph transcription (Williams et al. 2005; Tanifuji et al. 2014b; Sanitá Lima and Smith 2017), some of the orphan sRNAs described herein could also be intermediates of posttranscriptional processing of polycistronic mRNAs. However, such products would be expected to have low stability, to be present as short fragments, and/or to be underrepresented in RNA-seq data sets (Kuznetsova et al. 2017). This is not the case for the nucleomorph orphans we have identified. In fact, the *G*. *theta* orphans are the longest sRNAs represented in our libraries, and they have similar TPM counts as snoRNAs and snRNAs (supplementary table 6, Supplementary Material online). GtNM-R20 and GtNM-R21 correspond to sRNAs of 150 and 171 nt, respectively, compared with U1 snRNA, which at 118 nt is the longest nucleomorph sRNA of known function in *G*. *theta*. An intriguing observation comes from recent studies of nuclear noncoding transcription, which show that it is the act of transcription itself that can be important, rather than the function(s) of the noncoding RNAs that are generated (Ard et al. 2017). In other words, RNA transcription can serve to regulate the expression of neighboring protein-coding genes. It is thus possible that some of the pervasively transcribed nucleomorph genomic regions observed here and elsewhere (Tanifuji et al. 2014b; Sanitá Lima and Smith 2017) could serve such general regulatory functions.

### Light/Dark-Regulated sRNAs

The diurnal shift between light and darkness has major impacts on cell physiology and gene expression in photosynthetic organisms (Hirakawa et al. 2011; de los Reyes et al. 2017). To examine the influence of the light–dark cycle on nucleomorph sRNA expression, we extracted sRNAs from cells collected halfway into the respective light/dark phase of the diurnal cycle. In *G*. *theta*, only one nucleomorph sRNA was found to be significantly differentially expressed (|log_2_-fold change| ≥ 1.3, FDR<0.05). This was the orphan sRNA GtNM-R17, which was induced 6-fold under darkness (supplementary table 6, Supplementary Material online). Elucidating the role of this sRNA will require further investigation, as will the question of whether it belongs to any previously described sRNA class.

In *B*. *natans*, no nucleomorph sRNAs were found to accumulate to significantly different levels during the light–dark cycle (FDR>0.19 for all sRNA loci). Combined with the findings from *G*. *theta*, these results are consistent with the idea that the vast majority of nucleomorph sRNAs, including all snoRNAs and snRNAs, are constitutively expressed. This is contrary to the situation in animals, where snoRNAs show differential expression in, for example, different cancer types and in response to the circadian clock (Aitken and Semple 2017; Gong et al. 2017). Spliceosomal RNAs are differentially regulated under development, as observed in organisms as diverse as *Xenopus*, *Dictyostelium*, and pea plant (Lund and Dahlberg 1987; Hanley and Schuler 1991; Hinas et al. 2006).

The finding that almost all nucleomorph sRNAs investigated herein are nonresponsive to light–dark treatment might be unexpected for two photosynthetic organisms, but is in fact in line with a recent analysis of gene expression in *B*. *natans* by Suzuki et al. (2016). These authors found that nucleomorph protein-coding genes are constitutively expressed during the light–dark cycle, while 36% of nuclear mRNAs show differential expression. Among the differentially accumulated nuclear mRNAs were many that code for proteins targeted to the nucleomorph. Together with our sRNA data, these observations suggest that nuclear processes have taken over control of the diurnal response in the chlorarachniophyte nucleomorph.

### RNA Transport into the Nucleomorph?

A number of functional RNAs appear to be missing from the nucleomorphs of cryptophytes and chlorarachniophytes (e.g., H/ACA snoRNAs, RNase MRP and P RNAs, cryptophyte U5 snRNA). Nuclear orthologs exist for these RNAs, some of which could conceivable be imported from the host compartment. Dual protein targeting to the cytoplasm and PPC has been demonstrated for nuclear-encoded aminoacyl tRNA-synthetases (aaRSs) in chlorarachniophytes (Hirakawa et al. 2012a) and given that nucleomorph genomes lack genes for certain tRNAs, at least some RNAs are imported into the PPC and plastid (Douglas et al. 2001; Gilson et al. 2006).

From a mechanistic perspective the process of RNA transport from the host cytoplasm into the PPC/nucleomorph is not trivial. In both cryptophytes and chlorarachniophytes, PPC targeting means that two membranes would need to be crossed, that is, the outermost ER membrane and the periplastid membrane (Gould et al. 2006; Hirakawa et al. 2012b). Once inside the PPC, snoRNAs and snRNAs would need to further travel across the nucleomorph membrane via the nuclear pore complex. It is not known whether PPC/nucleomorph-destined RNAs would be transported “naked” or protein-bound. RNA is however highly susceptible to degradation by cellular RNases, and is thus typically transported protected in RNP complexes or enclosed in membranous vesicles (Jansen et al. 2014; Knip et al. 2014). In this case, we envision that cytoplasmic and periplastidial proteins would bind RNA on each side of the ER-periplastid membrane interface. The translocation process itself is complicated by the fact that nuclear-encoded, nucleomorph-targeted proteins are imported cotranslationally. Any putative RNA translocon would therefore most probably be encoded by the nucleomorph and/or plastid genome. Exploring the mechanisms of PPC/nucleomorph RNA import and characterization of the cytoplasm-to-PPC RNA translocation apparatus is an important future research topic.

## Conclusions

We have explored the complement of functional sRNAs in the nucleomorph genomes of the cryptophyte *G*. *theta* and the chlorarachniophyte *B*. *natans*. In the chlorarachniophyte, we found evidence for considerably shrunken U1 and U5 snRNAs. Most likely, these two RNAs have coevolved with the nucleomorph’s miniscule 18–21 nt introns. The *G*. *theta* nucleomorph encodes C/D box snoRNAs, which share rRNA 2′-*O* methylation targets with plant and animal snoRNAs. In summary, the nucleomorph has retained critical RNPs that constitute the RNA processing/modification and protein synthesis machineries, but a number of typical eukaryotic RNAs are missing. Nucleomorph sRNAs thus display the hallmarks of endosymbiosis: a reduction in molecular components and, presumably, EGT to the host genome.

## Supplementary Material

Supplementary data are available at *Genome Biology and Evolution* online.

## Supplementary Material

Supplementary Data

## Notes

**Data deposition:** The sRNA sequence data generated in this study have been submitted to NCBI’s Gene Expression Omnibus (GEO), series record GSE124831 (https://www.ncbi.nlm.nih.gov/geo/query/acc.cgi?acc=GSE124831; last accessed March 27, 2019). Novel sRNA annotations have been submitted to Genbank under accession numbers MK388843-MK388854.
