# Supplementary material for: Nucleomorph Small RNAs in Cryptophyte and Chlorarachniophyte Algae
Source: Genome Biol Evol. 2019 Apr 5;11(4):1117–34. doi: 10.1093/gbe/evz064 (PMC6461891; doi:10.1093/gbe/evz064)
Supplement: Supplementary Data [file evz064_supp.zip › Supplementary_Tables_and_Figures-R1.pdf]

Supplementary Tables.

Supplementary Table 1. snoRNA orthologs predicted by CM analysis.

Query snoRNA	Rfam accession	Target snoRNA	E value
SNORD38	RF00212	GtNM-R5	2.9e-05
SNORD52	RF00276	GtNM-R6	0.0013
N/A	N/A	GtNM-R7	N/A
SNORD46<br>GlsR7	RF00218<br>RF02476	GlsR7<br>GtNM-R8	0.98<br>3.6
SNORD14	RF00016	GtNM-R9<br>GlsR2	0.002<br>0.076
SNORD1 (snoR38)<br><br>GlsR6	RF00213<br><br>RF02475	GtNM-R10<br>GlsR6<br>GtNM-R10	0.93<br>0.25<br>0.021
SNORD29	RF00070	GtNM-R11	0.011
N/A	N/A	GtNM-R12	N/A
N/A	N/A	GtNM-R13	N/A
N/A	N/A	GtNM-R14	N/A
N/A	N/A	GtNM-R15	N/A

Supplementary Table 2. Accession numbers of sequences used in this study.

**Description**[1]	**NCBI/RNAcentral**[2]
G. theta NUC genome	GCA_000315625
G. theta NM chromosomes	NC_002752, NC_002753, NC_002751
G. theta PL genome	NC_000926
G. theta MT genome	Curtis BA, et al. 2012. Nature 492(7427):59-65
B. natans NUC genome	GCA_000320545
B. natans NM chromosomes	NC_010004, NC_010005, NC_010006
B. natans PL genome	NC_008408
B. natans MT genome	HQ840955
G. theta NM LSU rRNA	URS00001547D2
G. theta NM SSU rRNA	URS00002A0D1B
G. theta NM 5.8S rRNA	URS0000481336
B. natans NM LSU rRNA	URS0000AB278D
B. natans NM SSU rRNA	URS00006E7B68
B. natans NM 5.8S rRNA	URS00004A8106
Homo sapiens LSU rRNA	U13369 (nt 7935-12969)
Homo sapiens SSU rRNA	X03205
Homo sapiens 5.8S rRNA	U13369 (nt 6623-6779)
Arabidopsis thaliana LSU rRNA	URS00008CA728 (nt 361-3759)
Arabidopsis thaliana SSU rRNA	URS000008172F
Arabidopsis thaliana 5.8S rRNA	URS00001E2C22
Giardia intestinalis LSU rRNA	URS00008CC2B6

[1]MT: mitochondrial, NM: nucleomorph, NUC: nuclear, PL: plastid.
[2]RNAcentral accession numbers are in the form URS0000XXXXXX.

Supplementary Table 3. sRNA read data mapped to the four *G. theta* genomes.

Library		Raw reads	Trimmed and quality filtered[1]	Genome-mapped[2]			
				Nuclear	Nucleomorph	Plastid	Mitochondrial
**Dark-1**	**F**	1,895,933	1,642,879 (87%)	844,998 (51%)	49,822 (3%)	8,311 (0.5%)	70,930 (4%)
	**R**	1,895,933	1,641,696 (87%)	537,212 (33%)	33,036 (2%)	5,753 (0.4%)	55,593 (3%)
**Dark-2**	**F**	2,289,807	2,077,359 (91%)	1,282,230 (62%)	47,919 (2%)	8,181 (0.4%)	62,901 (3%)
	**R**	2,289,807	2,067,518 (90%)	796,847 (39%)	36,502 (2%)	5,165 (0.3%)	49,313 (2%)
**Light-1**	**F**	2,090,637	1,829,120 (87%)	801,644 (44%)	66,251 (4%)	8,287 (0.5%)	64,810 (3%)
	**R**	2,090,637	1,829,579 (88%)	526,651 (29%)	44,697 (2%)	5,879 (0.3%)	49,005 (3%)
**Light-2**	**F**	2,183,966	1,824,320 (84%)	862,761 (47%)	87,341 (5%)	9,051 (0.5%)	89,492 (5%)
	**R**	2,183,966	1,821,723 (83%)	592,852 (33%)	65,977 (4%)	6,515 (0.4%)	71,159 (4%)
**Dark-TE**	**F**	1,809,216	1,507,471 (83%)	660,584 (44%)	36,493 (2%)	9,055 (0.6%)	83,596 (6%)
	**R**	1,809,216	1,510,564 (83%)	433,715 (29%)	23,647 (2%)	6,201 (0.4%)	63,235 (4%)
**Light-TE**	**F**	2,433,723	1,930,984 (79%)	723,433 (37%)	34,093 (2%)	9,122 (0.5%)	67,299 (3%)
	**R**	2,433,723	1,933,927 (79%)	548,675 (28%)	26,569 (1%)	6,847 (0.4%)	53,808 (3%)
**neg**[3]	**F**	203,836	9,617 (5%)	1,495 (16%)	119 (1%)	12 (0.1%)	119 (1%)
	**R**	203,836	42,097 (21%)	662 (2%)	48 (0.1%)	5 (0.01%)	60 (0.1%)
**pos**[4]	**F**	3,240,545	3,195,961 (99%)	1,304 (0.4%)	46 ($1e^{-3}$ %)	7 ($2e^{-4}$ %)	56 ($2e^{-3}$ %)
	**R**	3,240,545	3,174,800 (98%)	1,276 (0.4%)	30 ($9e^{-4}$ %)	2 ($6e^{-5}$ %)	34 ($1e^{-3}$ %)

[1]Trimmed reads >15 nt after adapter removal.

[2]Percentage of trimmed and quality filtered reads mapping to each genome. Mapping was done using PatMan in the UEA sRNA workbench.

[3]neg: negative technical control (no template in reverse transcription).

[4]pos: positive technical control (microRNA from *Arabidopsis thaliana*).

Supplementary Table 4. sRNA read data mapped to the four *B. natans* genomes.

Library		Raw reads	Trimmed and quality filtered[1]	Genome-mapped[2]			
				Nuclear	Nucleomorph	Plastid	Mitochondrial
Dark-1	F	2,119,216	1,019,667 (48%)	302,126 (15%)	4,941 (0.2%)	9,988 (0.5%)	588 (0.03%)
	R	2,119,216	1,045,082 (49%)				
Dark-2	F	2,416,930	1,805,544 (75%)	311,302 (9%)	7,403 (0.2%)	13,465 (0.5%)	611 (0.02%)
	R	2,416,930	1,764,051 (73%)				
Dark-3	F	2,742,102	848,137 (31%)	214,098 (12%)	2,980 (0.2%)	8,995 (0.4%)	527 (0.02%)
	R	2,742,102	943,878 (34%)				
Light-1	F	2,476,394	1,807,627 (73%)	374,644 (10%)	8,154 (0.2%)	19,165 (0.7%)	549 (0.01%)
	R	2,476,394	1,772,982 (72%)				
Light-2	F	2,128,540	1,728,771 (81%)	274,354 (8%)	6,855 (0.2%)	23,571 (0.5%)	432 (0.03%)
	R	2,128,540	1,673,959 (79%)				
Light-3	F	2,586,247	896,381 (35%)	265,809 (14%)	4,462 (0.2%)	12,432 (0.7%)	307 (0.02%)
	R	2,586,247	984,297 (38%)				
pos[3]	F	3,175,863	3,108,503 (98%)	468 ($8e^{-3}$ %)	6 ($1e^{-4}$ %)	10 ($2e^{-4}$ %)	0 (0)
	R	3,175,863	2,979,179 (94%)				

[1]Trimmed reads >15 nt after adapter removal.

[2]Percentage of trimmed and quality filtered reads mapping to each genome. Mapping was done using Bowtie2. The forward and reverse sRNA libraries were merged before mapping.

[3]pos: positive technical control (microRNA from *Arabidopsis thaliana*).

Supplementary Table 5. Sequences of *G. theta* nucleomorph sRNAs discovered or confirmed in this study.

RNA	Functional assignment	Sequence	Length nt	Over-lap (nt)[2]
GtNM-R1	U1 snRNA	AUACUUACCUAGCAUUCAAAAAUAACUAAAGUUAUUUUUCAAUAUAUUGUAUCUGCUUCACAUUGAUUUGAUAUUUUGGC UCUAAUUGUUAAGAAUGCUAAAUUUUUUUUACUGUGG	118	3’ (9)
GtNM-R2	U2 snRNA	AUAGUAGAUUUUAUGCUAAGAUCUUAAUGUAAAUUACUUAUUUCAGUAACAUCUGAAUAAUUACUUUUUAAAUUUUUUUU UUGUUAUUCACCCAAUACUUGUGGAU	107	no
GtNM-R3	U4 snRNA	AUUCUUACGUAGAGGUAUUCUUUUCAAGCGAUAACUGAUGAAAAGAGCUUUUGCAUUUUGAAAACUUUAAUUUAGAUAAUU UUUUAAAAUC	91	3’ (2)
GtNM-R4	U6 snRNA[1]	GGAUAUUCCAUCAUAAAUUUAAUACAAUACAGAGAAGAUUAGCAUGGCCCCUGCGUAAGGAUGACAUGAAAAUCUACUGGU AGUAUUUU	89	no
GtNM-R5	C/D box snoRNA (snR1[1])	AGCUAAUGAUGAAAAUUUAAUCCGGUUCUGCUUUUGAUUAAUUGAUAAAAUUUCUCUGACAGCUG	65	no
GtNM-R6	C/D box snoRNA (snR2[1])	AUAAAAUGAUGCAGACUAGAGUCUUUGAUAUAUUUGACAAUAAUUAUUACUGAAAU	56	no
GtNM-R7	C/D box snoRNA (snR3[1])	AUAUAAAUAAAUGAUGAUAGUUUGACGGUCUAAUGAUAUCAAUGAAAAUCAAUUCACCUGAACU	65	3’ (2)
GtNM-R8	C/D box snoRNA (snR4[1])	UCACAUGAUGACAACUAAUUAAAUGAAAUUAUUAAUGACACUUAACCUCAUAGUUACACUGAUUAA	66	3’ (4)
GtNM-R9	C/D box snoRNA (snR5[1])	UUGCUAUGAUGAUUUGCUCCUCAACUGUUCGCUUAUAAAUUCUUGAAAAAUUCUACUAUAAGAAUUAUUCGUAAGUAAUCC UUGGAUGUCUGAGCUUU	98	no
GtNM-R10	C/D box snoRNA	GUGAAAUUUUAAUGAUGUUGUUAUCCCUGUAUGAAAUUUUGAUUGUUCAAAUCUGACUUACUU	63	3’ (6)
GtNM-R11	C/D box snoRNA	ACAACUAUGAUGUUCUUAGCUCACAUUGAUUAAUGAUUAAAUCAUAUAAACUGAGCUUUGUUAGUAU	67	no
GtNM-R11- Ψ1	Pseudogene C/D box snoRNA	ACAACUAUGAUGUUCUUAGCUCACAUUGAUUAAU	34	no
GtNM-R11- Ψ2	Pseudogene C/D box snoRNA	ACAACUAUGAUGUUCUUAGCUCACAUUGAUUAAU	34	no
GtNM-R12	C/D box snoRNA	AAUUAAAAUUAUGAUUAUAAUGACGGUAUCUGAAGUAAUAUGAUAAUAACAGACCUAUAAUGAUUUAU	68	3’ (14)
GtNM-R13	C/D box snoRNA	UUUUAAUGAUUAUUAUUUGUGCUGAAUUUCUUGAAAUAUUCAUCUUUCGAAUGAAUAAU	59	no
GtNM-R14	C/D box snoRNA	AAAUUUAAAUGAUUAAAAGUGGGCGGAAUGAUAUUUGAUUAAAUUUACUAAUUGAUUAUGAAU	63	no
GtNM-R15	C/D box snoRNA	AUACUGUGAAGAAAUAUGGCAUUUGUAUGAAUAAAUUGAUUUAUAGAACACACUGAUUUUUAACAAAU	68	no
GtNM-R16	Orphan RNA	AUGUUAAACAAUUGAUUCUUCGCAGUAUUGAUAGAAUAUGAUUUGUGAGUUGGUUAUGAAACAUGAUCUGUAAAAAAUGAU UCU	84	no
GtNM-R17	Orphan RNA	AAUGAAAUAUAUAGUAUAAUUUGUAGAUUUGACAAAUAAAUACAAAAAUAUAAAUUAUAAAAAUGACAAAUUUGUAUAAG	80	3’ (18)
GtNM-R18	Orphan RNA	AGUAUAUACUCCCACCAAAAAAAUGAUGCUUGUGUCUGUGUUAAUGAAUUUGAUAAUGAAAGUAUCUGAUU	71	no
GtNM-R19	Orphan RNA	UUUAUAUUAAAUUAAAAUUUAACUAAAUUAUAUUUUAAAUUAAUUUAAACUUUUUUAUAAAUUUUAAAUUUGUAAUUUUAUA AUGAAAAUUUGGU	95	3’ (13)

GtNM-R20	Orphan RNA	AGUUUUUUCAAAUGUAACAUUUUGUAAAUAAAAAUAUAAUAUAUUUUUUAAUUUAAAUUAUUACCAAGUUAUAGUUUUUUUU UCAAAUUGCAGUUAAAAUUUGAUGAUGGGUUUAUUUAUCAUCAAUAAAAGUAAUUUGAAUAAAAUUUU	150	5’ (20)
GtNM-R21	Orphan RNA	GACUGUACUUGUACAGGAUCUUAAAAUGAGUAAAAGCUCACACGCAUAUCUAUAUAAAGUUUUAUGAUUAAAAUGUUAUUU AACACAGAUAUUGUUCACAAUUUUAUGUAUAAAAUUAAAAUGAAAACUUAUUUCAGAUUUAAUUCUUGCUUUAUAAAGUUGC AGGUACUC	171	3’ (43)

[1]Douglas et al. 2001.
[2]Overlap between the RNA transcript and a neighboring protein-coding gene at the 5’ or 3’ end.
C, D’, C’ and D box elements of C/D box snoRNAs are shaded in grey.

Supplementary Table 7. *B. natans* RNAs discovered or confirmed in this study.

RNA	Functional assignment	Sequence	Length nt	Over-lap (nt)[2]
BnNM-R1	U1 snRNA[1]	ACUUACCUGGUCGGGAAAUUGUUCUGAAUUUUCUUUAACUAAUCUAAGUUCAGGGAUUUUAUAUACCCACGACA UAAUUUUUGCAGGAUUUCUGCUAUUAAA	102	no
BnNM-R2	U2 snRNA[1]	UACCUAUCUCGGCCUUUUGGCUAAGAUCAAGUGUAGUAUCUGUUCUUAGCAACUUAACCGUUGCUAUGAGAUUC AAAGAAUCUCGUCUCAAUUUUUACUAAGGCUUUACCAAUUAAGGAAUUCGGCCUUG	130	no
BnNM-R3	U4 snRNA	ACUUGCGCUUGGGGACAAACAAUCUCAAAUGAUUUUCUUGAUGAGAUUGAAUGCUAGUUGAAAAUAAUUCCGCU UAACUUAUUUUUACCCCUCAUU	96	no
BnNM-R4	U5 snRNA[1]	AUCUAUUCUUUCGCCUUUUACUAAAGAAUACUUAAAAUAAGAGCACAAUUUUUGCUUC	58	3’ (2)
BnNM-R5	U6 snRNA[1]	GCGUUCACGCAUCUUUAAAAUUGGAACGAUACAGAGAAGAUUAGCAUGGCCCCUGCGCAAGGAUGACACGCAUA AUCCGAGAUUAGGUUCCUUUAUUU	98	no
BnNM-R6	5S rRNA	AGUAACGGUCAUACAAGUUGAAAACAACCUUAAAUCCCUCAGACCUAAGAAGUUGAAAACAGCUUUGGCAGUGUG AGUACUAGUUUGAGCAAUCGACUGGGAAAGCAUUGUACCGUUGCUUU	122	no
BnNM-R7	tRNA-Phe^{GAA}	AAUUGGGGUUGUAGCUCAGCUGGUAGAGCGUUAGACU**GAA**GAUCUAAAGGUCAUUAGUUCAAAACUAAUCGAUC CCAU	78	no
BnNM-R8	C/D box snoRNA-like	UGAACUGAUUAUAUAGGUCUGUGUAAUGAUUAGAUAUUACAAUCAUGAUUUAUUU	55	3’ (6)
BnNM-R9	Orphan RNA	AUGAUGGGAUCUUAUCCUAAUUAUGAAAUGGAUGAAAGUAUCAUAAAG	48	no
BnNM-R10	Orphan RNA	AUGAUACACAAUAAAAAAUAUUUUGACAUUUUAUUUUUAUUCAAUGAUAAAAAAUUUUUCAAC	63	no
BnNM-R11	Orphan RNA	AUGAGAAGAAAUAGCAAUUCUCAUUCUGAAAAUGUAAUAAUAAAAUAAUAAGAUACUAUUAAAUAUAGUUAGAU	74	no
BnNM-R12	Orphan RNA	AUAACAUGAUUGCAUGGCUCAUUUUUUUUUAUAAUAUUUAUUUAUGAUACUAAUUCUAAU	60	no
BnNM-R13	Orphan RNA	ACGACCUUACUUUAAAGGAUCAUAGUAGUUGUUUUUUCCUGUAACAUUAAACCAAUGAUGAAUAUCCAACUGAGG UUUUAGAAAUUCAUGCAUU	94	no
BnNM-R14	Orphan RNA	UAGUAUAAAUGGGUUAAGUUAUUUAUUAUAGGAAUCUCAAGAAGUAACAUG	51	3’ (3)

[1] Gilson et al. 2006.
[2]Overlap between the RNA transcript and a neighboring protein-coding gene at the 3’ end.
C/D box-like elements in orphan RNAs are in shaded grey.

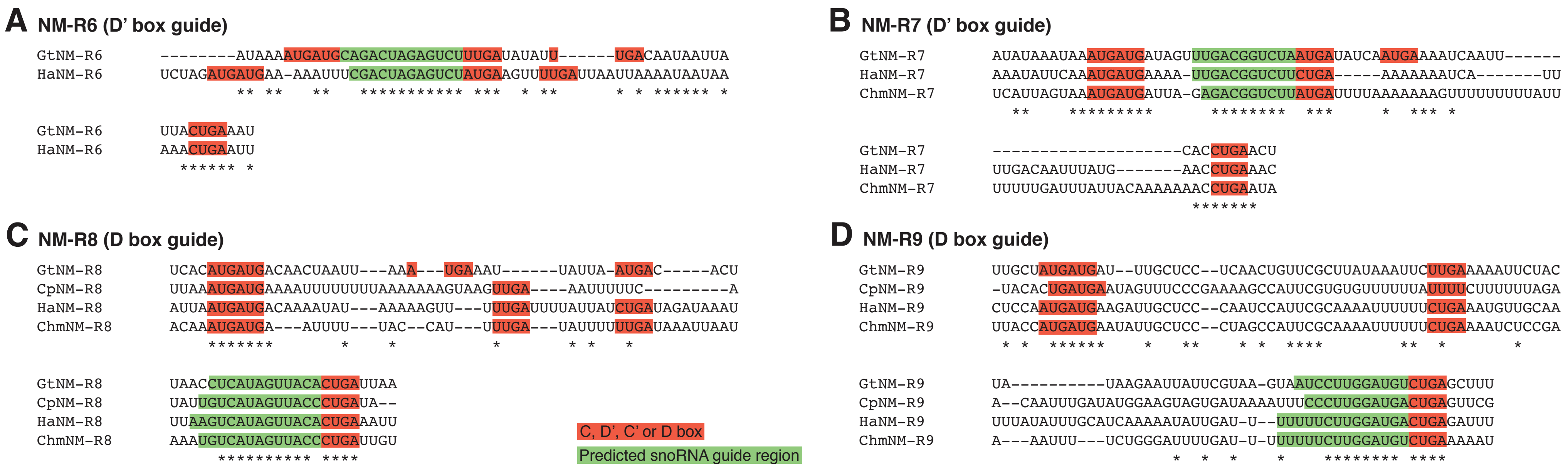

Supplementary FIG. 1. The guide regions of snoRNAs GtNM-R6–9 are conserved in four cryptophyte nucleomorph genomes. Guide regions were predicted by Plexy (Kehr et al. 2011, *Bioinformatics* 27:279-280) and are highlighted in green background. C, D', C' and D boxes are highlighted in red. Chm: *Chroomonas mesostigmatica*, Cp: *Cryptomonas paramecium*, Gt: *Guillardia theta*, Ha: *Hemiselmis andersenii*, NM: nucleomorph.

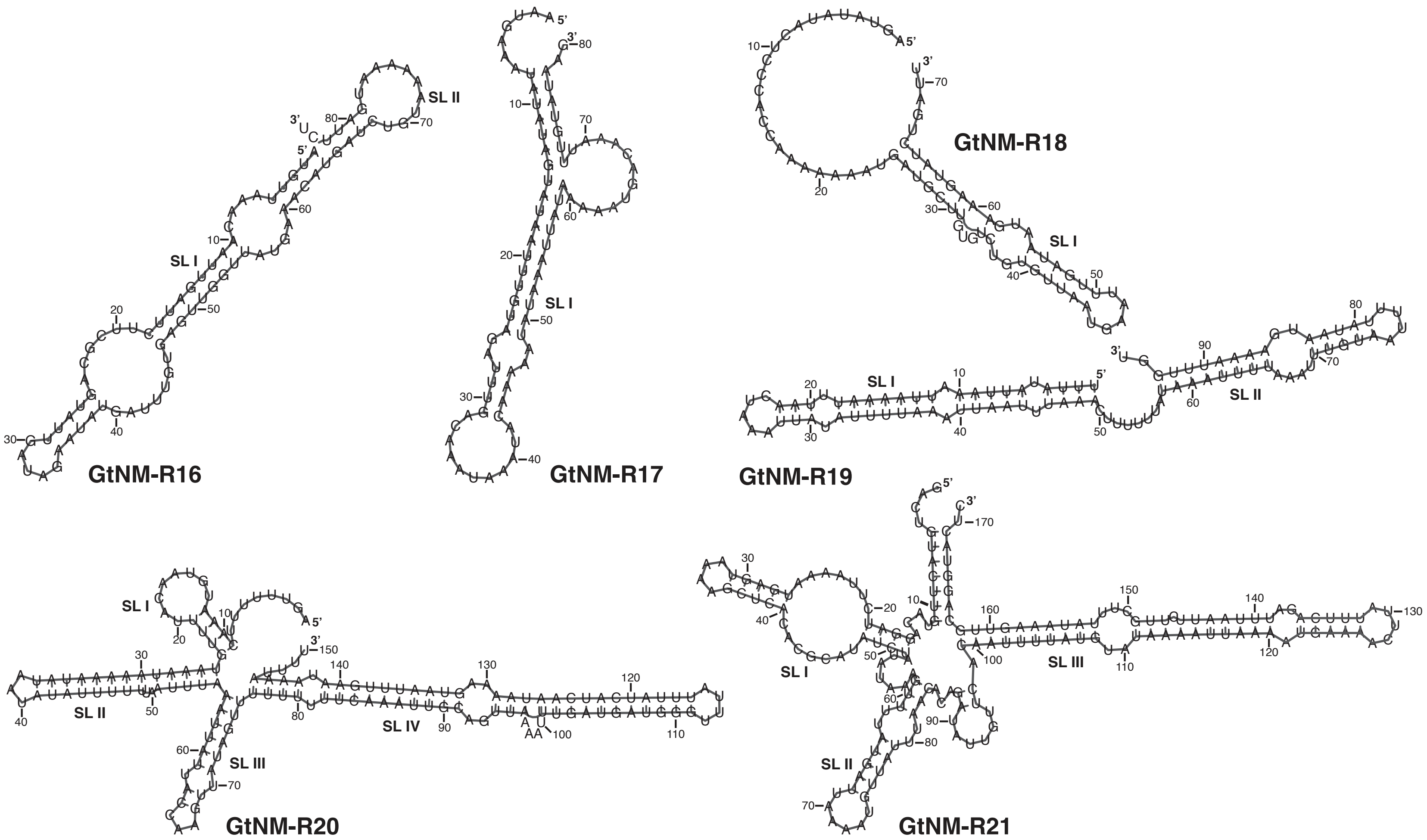

Supplementary FIG. 2. **Secondary structures of orphan sRNAs from the *Guillardia theta* nucleomorph.** Homology searches and secondary structure prediction did not reveal any obvious functions for GtNM-R16–21.

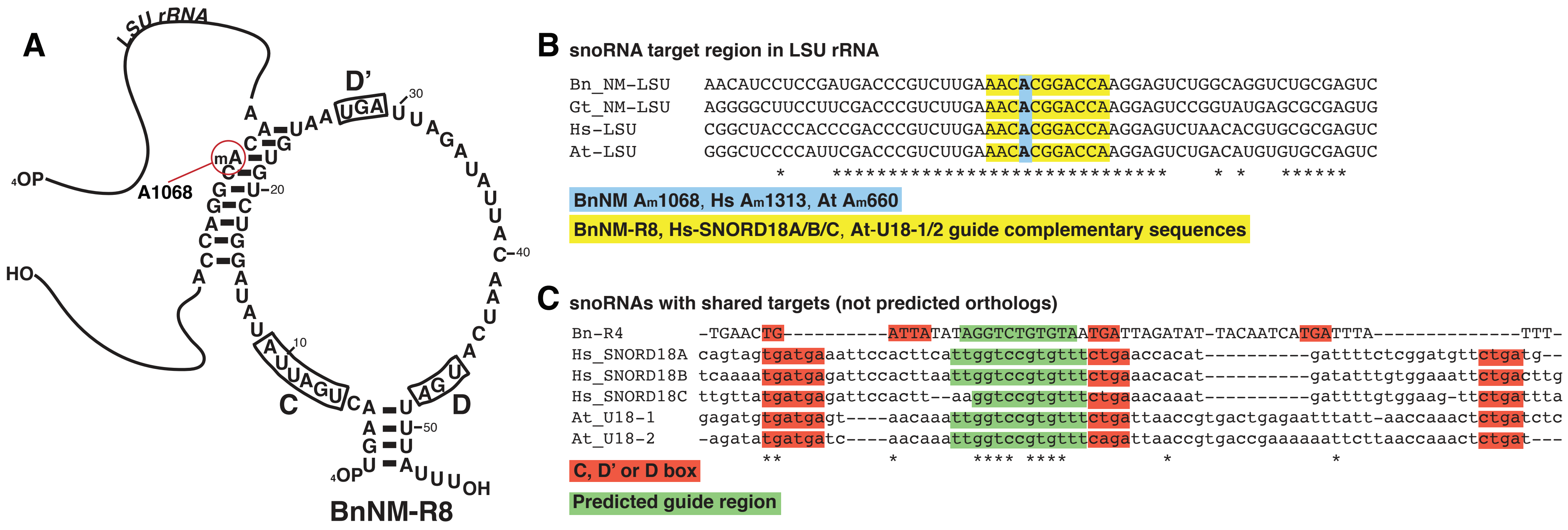

Supplementary FIG. 3. C/D box snoRNA-like BnNM-R8 shares predicted rRNA target region with *Arabidopsis* and human. **A** Secondary structure model of BnNM-R8 and its interaction with nucleomorph LSU rRNA. The putative D' guide region and the predicted 2'-*O* methylation target position A1068 are indicated. **B** BnNM-R8, human SNORD18 and *Arabidopsis* U18 have identical LSU targets. **C** Sequences of BnNM-R8 and plant/animal SNORD18 homologs. Guide regions in human and *Arabidopsis* were obtained from the snOPY database (Yoshihama et al. 2013, *BMC Res Notes* 6). Highlighted in green: the predicted guide region of nucleomorph BnNM-R8 and the 2'-*O* methylation guides of human and *Arabidopsis* SNORD18. C, D' and D boxes are shown in red background.

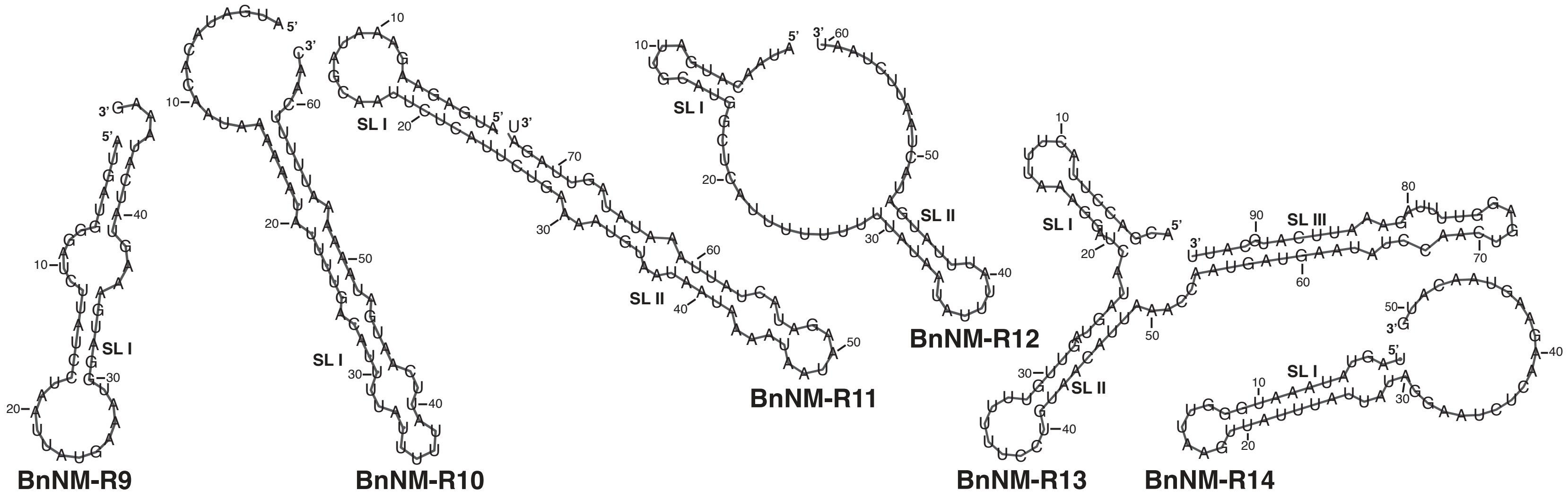

Supplementary FIG. 4. Secondary structures of orphan sRNAs from the *Bigelowiella natans* nucleomorph. Homology searches and secondary structure predictions failed to reveal any functions for BnNM-R9–14.
