# Supplementary material for: Nucleomorph Small RNAs in Cryptophyte and Chlorarachniophyte Algae
Source: Genome Biol Evol. 2019 Apr 5;11(4):1117–34. doi: 10.1093/gbe/evz064 (PMC6461891; doi:10.1093/gbe/evz064)
Supplement: Supplementary Data [file evz064_supp.zip › Suppl_legends-R1.doc]

**Supplementary legends**

**Supplementary FIG. 1**. **The guide regions of snoRNAs GtNM-R6−9** **are conserved in four cryptophyte nucleomorph genomes.** Guide regions were predicted by Plexy (Kehr et al. 2011, *Bioinformatics* 27:279-280) and are highlighted in green background. C, D’, C’ and D boxes are highlighted in red. Chm: *Chroomonas mesostigmatica*, Cp: *Cryptomonas* *paramecium*, Gt: *Guillardia* *theta*, Ha: *Hemiselmis* *andersenii*, NM: nucleomorph.

**Supplementary FIG. 2. Secondary structures of orphan sRNAs from the *Guillardia* *theta* nucleomorph.** Homology searches and secondary structure prediction did not reveal any obvious functions for GtNM-R16−21.

**Supplementary FIG. 3**. **C/D box snoRNA-like BnNM-R8 share predicted rRNA target region with *Arabidopsis* and human. A** Secondary structure model of BnNM-R8 and its interaction with nucleomorph LSU rRNA. The putative D’ guide region and the predicted 2’-*O* methylation target position A1068 are indicated. **B** BnNM-R8, human SNORD18 and *Arabidopsis* U18 have identical LSU targets. **C** Sequences of BnNM-R8 and plant/animal SNORD18 homologs. Guide regions in human and *Arabidopsis* were obtained from the snOPY database (Yoshihama et al. 2013, *BMC Res Notes* 6). Highlighted in green: the predicted guide region of nucleomorph BnNM-R8 and the 2’-*O* methylation guides of human and *Arabidopsis* SNORD18. C, D’ and D boxes are shown in red background.

**Supplementary FIG. 4. Secondary structures of orphan sRNAs from the *Bigelowiella* *natans* nucleomorph.** Homology searches and secondary structure predictions failed to reveal any functions for BnNM-R9−14.

**Supplementary Table 1. snoRNA orthologs predicted by CM analysis.**

**Supplementary Table 2. Accession numbers of sequences used in this study.**

**Supplementary Table 3. sRNA read data mapped to the four *G. theta* genomes.**

**Supplementary Table 4. sRNA read data mapped to the four *B. natans* genomes.**

**Supplementary Table 5.** **Sequences of *G. theta* nucleomorph sRNAs discovered or confirmed in this study.**

**Supplementary Table 6. sRNA read count (TPM) and differential expression analysis.**

**Supplementary Table 7.** ***B. natans*** **RNAs discovered or confirmed in this study.**
