# Supplementary material for: Nucleomorph Small RNAs in Cryptophyte and Chlorarachniophyte Algae
Source: Genome Biol Evol. 2019 Apr 5;11(4):1117–34. doi: 10.1093/gbe/evz064 (PMC6461891; doi:10.1093/gbe/evz064)
Supplement: Supplementary Data [file evz064_supp.zip › Supplementary_data1.doc]

**Revised annotation of nuclear *u1a*/*u2b***

Nucleotide and amino acid sequences of the predicted nuclear-encoded, nucleomorph-targeted U1A/U2B in *G*. *theta*.

>Guith1|scaffold_70:308631-310358

ATGGGGGCAGTGGGGTTGGTAGTCGTGTTGTCTCTGGTATCGCAGCTTCATGGTTGCGGTGTTGATTCCGTGCAATGCGCTGGTCTCACTTCTCACGCACACGCGCCCTTTCTGAGCCTTCGGGGAGGGTCCAGTTCGGACGAGATGGATCTGAAGTTTTCGGAGGAGGAGACCATGTCTTTGGGGAGAGACACAGAATCGGATGCGATGAGGATAGAGCAGAACTATGGCGTTGAGGTCCCACGAGCCCTCATGGGCCATCGACAACTGGTTAGTGACGTCACGGGGCCGCAATATCTCAGGAGAAAGGATGGCGAGATCGTTGCGGACAACGGCATCGACTCGGAGCACAGGCATCCGTACCGCTACAAGGAATCTTACGACCAGCTTCCACCATGCAACACCCTCTACATCAGGAACTTGAGGGAGCGTGTGAAACCTAGGCGGATGCGAGAGCTCTTGTACGCTGTGTTCTCCCAGTACGGCAAGATCCTCGACGTAAGGGCGCTCAAGACGCAGAAGATGCGAGGACAGGCCTTCGTGTGCTTCGACGAGCTGAAGGCGAGGGAGCAGCTGGAACATGTGCAGGGCCAGTCGATGCTGACGCCATGTGCTTGTCAGGATGCTGTGCATGCTAAGGAGGAGCTGAACGGATTTTCATTCTTGAACCGGACGCTAGTCATCCAGTTTGCCAAGAGTCCGAGTCATGCACACCTCAAGAAGGATGGAACTTACTCAGAGATGATGCAGCAGATCCGATCTGAGTACTGGTCCAATCACAGTTTGACTATCAGGAAGTCTGTGGAAACCGCCCTGGAGTCGAAGGCGAACACCTCTTCCGTCGCTCAGCTCAACATCCTCGAGGACGACTGGCTCTTGTCGCAACACGGCTATAAGCTGAGCAAGGAGCCCTCTCGCCATCTCCTCATCACCAACTGCCCTGCCAACATCATCGAGCAGGACGTTTTGGATGTCCTCGGGCAGTTCGAGGGGCTGATCTCCGTGGAGAAGATCTCGGGAGCTGTTGCGTTCATGGCTCACTACGAGGAGAAGGATACTGCGTCGGAGGCGACGGAGGCGCTGGAGGGATTCTTCTTTCCCGAGGAGCCGGACTTCATCATCCGCCTACACTACATCACCTCCCTTCACCAATGA

>113851

MGAVGLVVVLSLVSQLHGCGVDSVQCAGLTSHAHAPFLSLRGGSSSDEMDLKFSEEETMSLGRDTESDAMRIEQNYGVEVPRALMGHRQLVSDVTGPQYLRRKDGEIVADNGIDSEHRHPYRYKESYDQLPPCNTLYIRNLRERVKPRRMRELLYAVFSQYGKILDVRALKTQKMRGQAFVCFDELKAREQLEHVQGQSMLTPCACQDAVHAKEELNGFSFLNRTLVIQFAKSPSHAHLKKDGTYSEMMQQIRSEYWSNHSLTIRKSVETALESKANTSSVAQLNILEDDWLLSQHGYKLSKEPSRHLLITNCPANIIEQDVLDVLGQFEGLISVEKISGAVAFMAHYEEKDTASEATEALEGFFFPEEPDFIIRLHYITSLHQ*

Green: manually annotated start codon/initiation methionine.

Yellow: currently annotated start codon/initiation methionine.

Blue lettering: N-terminally extended sequence encoded from an alternative start codon.

Predicted SP cleavage site: between positions 18 and 19, LHG-CG. The TP does not initiate with the aromatic amino acid needed for targeting to the complex plastid. The protein is therefore predicted to have PPC/nucleomorph localization (Gould et al., 2006, Gruber et al. 2007).

Grey: highly similar to RNA recognition motifs RNP2 and RNP1 in human U1A, GenBank NP_004587.1 (Oubridge *et al*., 1994).

**Novel sRNAs from the *G. theta* nucleomorph aligned with snoRNAs from *Arabidopsis* and human.**

Green: guide sequences; pink: C, D’, C’ and D boxes; At: *Arabidopsis thaliana*; Hs: *Homo sapiens*; GtNM: *G. theta* nucleomorph. Clustal Omega 1.2.4 was used.

```
GtNM-R12

GtNM-R12        AAUUAAAAUUAUGAUUAUAAUGACGGUAUCUGAAGUAAUAUGAUAAUAACAGACCUAUAA	60
HsSNORD25       --UUCCUAUGAUGAGGAC------------------------CUUUUCACAGACCUGUAC	34
AtSnoR19-1      ---AACAGUGAUGAGU--------------------------CAGUUUACAGACCUGUAA	31
                        * ****                                * ******** **
GtNM-R12        UGAUUUAU---------------------------------------	68
HsSNORD25       UGAGCUCCGUGAGGAUAA-AUAACUCUGAG-------GAGA------	67
AtSnoR19-1      UGAUUGCGGUAAUGAUCGCAUUAUUAUGAACAUCUAAGGGACUGAGU	78
                ***


GtNM-R13

GtNM-R13       -------------------------UUUUAAU-----------GAUUAUUAUUUGUGCUG	24
HsSNORD51      -------------------------------------------------GUUGCAUGAUG	11
AtU51A         GCAAUCAGUGAUGAAAUUAUGUCAAAUUCAAUGAUUAUACAAUGAUUAUUAUA-------	53
                                                                  *
GtNM-R13       AAUUUCUUGAAAUAUUCAUCUUUCGAAUGAAUAAU------------------------	59
HsSNORD51      AAUAAAAUCAAAUCACCAUCUUUCGGCUGAGUUCGUGAUGGAUUUGCUUUUUUCUGAUU	70
AtU51A         -A--UCACUAGAUCACCAUCUUUCGGCUGAGAUUGC-----------------------	86
                *       * **   *********  ***


GtNM-R14

GtNM-R14      AAAUUUAAAUGA---------UUAAAAGUGG-----------------GCGGA---AUGA	31
AtU40-2       ---UCCUAAUGAGGAUAUUUAUCGAAAGCUAUGGAUCUGUUUAUCAGUUCCAUAUGAUGC	57
                 *   *****         *  ****                     *      ***
GtNM-R14      UAUUUGAUUAAAUUUACUAAUUGAUUAUGAAU--	63
AtU40-2       UAUAUUGUCAUAGUUACU--------CUGAUGGG	83
              *** *  * * * *****         ***


GtNM-R15

GtNM-R15         --AUACUGUGAAGAAAUA------------------------------------------	16
Hs_SNORD15A      --CUUCGAUGAAGAGA----UGAUGACGAGUCUGACUUGGGGAUGUUCUCUUUGCCCAGG	54
AtU15-1A         CUCAGUGAUGAAGAAACAAUAGAUGACGAGUCCG--------------------------	34
                         ****** *
GtNM-R15         ------------------------UGGCAU-------------UUGUAUGAA-UAAAUUG	38
Hs_SNORD15A      UGGCCUACUCUGUGCUGCGUUCUGUGGCACAGUUUAAAGAGCCCUGGUUGAAGUAAUUUC	114
AtU15-1A         -----------------------------------------------AUGAAAUCCAUUC	47
                                                                 **** *   **
GtNM-R15         AUUUAU--------AGAACACACUGAUUUUUA--ACAAAU	68
Hs_SNORD15A      CUAAAGA-----UGACU-UAGAGGCAUUUGUCUGAGAAGG	148
AtU15-1A         AUAAAAUCGUGGGGACAAACGAGGCAUUUGUCUGAGA---	84
```

* *	*	*	**** *	* *

**Chlorarachniophyte 5S rRNA**

Sequences were predicted by Infernal (Nawrocki and Eddy, 2013)

>AaNM_5S

CGUUACGGUCAUACUACUUGGAAAAUACCCUACUCCUUCAGAAUUAGGAAGUUAAAaACAAGUUAGGCAGCUUGAGUACUAUUCGAGAAAUCGAUUGGGAAAAUGUUGUACUGUGAUGC

>BnNM_5S

AGUAACGGUCAUACAAGUUGAAAACAACCUUAAAUCCCUCAGACCUAAGAAGUUGAAAACAGCUUUGGCAGUGUGAGUACUAGUUUGAGCAAUCGACUGGGAAAGCAUUGUACCGUUGCUUU

>LvNM_5S

CGUUACGGUCAUACAACUAGCAAUAAACCCUGUCACCUCAGACCCAGGAAGUAAAAACUAGUUUGGCAUGAUUAGUACUGAAUUGAGAAAUCGUUUGGGAAUUUCAUGUACUGUUACGC

>LoNM_5S

CGCAACGGUCAUACAACUAGCAAUGUACCCUGUAACCUCAGACCCAGGAAGUGAAACCUAGUUUGGCGUGUCUAGUACUGAAUUGAGCAAUCGUUCGGGAAUUUCAUGUACUGUUAUGC

Aa: *Amorphochlora amoebiformis* Bn: *Bigelowiella natans*, Lo: *Lotharella oceanica*, Lv: *Lotharella vacuolata*, NM: nucleomorph.

**References**

Gruber A, Vugrinec S, Hempel F, Gould SB, Maier UG, Kroth PG. (2007) Protein targeting into complex diatom plastids: functional characterisation of a specific targeting motif. Plant Mol Biol. Jul;64(5):519-30.

Gould SB, Sommer MS, Kroth PG, Gile GH, Keeling PJ, Maier UG. (2006) Nucleus-to-nucleus gene transfer and protein retargeting into a remnant cytoplasm of cryptophytes and diatoms. Mol Biol Evol. Dec;23(12):2413-22.

Nawrocki EP, Eddy SR. (2013) Infernal 1.1: 100-fold faster RNA homology searches. Bioinformatics. 29(22):2933-5.

Oubridge C, Ito N, Evans PR, Teo CH, Nagai K. (1994) Crystal structure at 1.92 A resolution of the RNA-binding domain of the U1A spliceosomal protein complexed with an RNA hairpin. Nature. 372(6505):432-8.
